# Walks with jumps: a neurobiologically motivated class of paths in the hyperbolic plane

**DOI:** 10.1007/s00285-026-02347-9

**Published:** 2026-02-13

**Authors:** Jason DeBlois, Eduard Einstein, Jonathan D. Victor

**Affiliations:** 1 Department of Mathematics, University of Pittsburgh, 301 Thackeray Hall, Pittsburgh, PA 15260 USA; 2 Swarthmore College Department of Mathematics and Statistics, 500 College Ave., Swarthmore, PA 19081 US; 3 Feil Family Brain and Mind Research Institute, Weill Cornell Medicine, New York, NY 10065 USA

**Keywords:** 51M10, 92C20

## Abstract

We introduce the notion of a “walk with jumps”, which we conceive as an evolving process in which a point moves in a space (here, $\mathbb {H}^2$) over time, in a consistent direction and at a consistent speed except that it is interrupted by a finite set of “jumps” in a fixed direction and distance from the walk direction. Our motivation is biological; specifically, to use walks with jumps to represent the activity of a neuron over time (a “spike train”). This representation has distinctive properties, including a built-in “point of no return” property that may serve as a substrate for decision-making with progressive refinement over time. Moreover, because (in $\mathbb {H}^2$) the walk is built out of a sequence of transformations that do not commute, the walk’s endpoint encodes aspects of the sequence of jump times beyond their total number. Importantly, this encoding is incomplete: quite different sequences of jump times may lead to the same endpoint. The main results of the paper use the tools of hyperbolic geometry to formalize and delineate the these behaviors.

## Introduction

### Biological Motivation

A central challenge of systems neuroscience is to understand and model how the aggregate activity of a network of neurons performs cognitive tasks, such as representing the external world or making decisions. A fundamental principle that guides attempts to relate the cognitive domain to that of neural activity is that similar percepts should correspond to similar patterns of activity (Edelman 1998; Kriegeskorte et al. 2008; Kriegeskorte and Kievit 2013). In a sense, this principle recasts the neuroscience question into a mathematical one: what are appropriate geometries or topologies by which neural similarity should be measured?

Here, we are motivated by a basic aspect of this question: what are appropriate mathematical models for similarity relationships between different patterns of activity of a single neuron? With the starting point of considering a sample of neural activity—a spike train—to be an instance of a point process, many such models are in common use. The simplest of these is a “spike count” code, in which the only relevant feature of a spike train is the number of spikes it contains. However, experimental evidence indicates that the arrangement of spikes in time is also behaviorally relevant (Di Lorenzo et al. 2009; Jacobs et al. 2009). This has motivated models in which the timing of spikes plays a role in quantifying similarity – either as estimators of a firing rate envelope (van Rossum 2001), or through their detailed pattern and/or interval structure (e.g. Victor and Purpura (1997)).

The goal of this paper is to introduce another model class—walks with jumps—and establish the mathematical underpinnings of its distinctive characteristics. The central aspect of these models is a correspondence between point processes and paths in the hyperbolic plane. With this correspondence, two spike trains can be compared based on the distance between their endpoints. This leads to the distinctive characteristics of the walks-with-jumps model, which emerge as consequences of the underlying hyperbolic geometry. While perhaps one can view these characteristics as intuitive, proving them is not as straightforward as might be expected. These proofs are the main substance of the paper; here, we want to give them some biological context.

One distinctive characteristic of the walks-with-jumps model, explored in Proposition 1.10 and Sections 4 and 5, is that of a “point of no return:” if two spike trains sharing an initial part have a sufficiently large gap between subsequent spikes, then their endpoints can never be the same (Theorem 1.8). While some aspects of this behavior are shared by other models, the behavior of the walks-with-jumps model is distinctive: the “point of no return” property has a surprising counterpoint: two spike trains can have identical endpoints, but differ substantially in the number (Proposition 3.4) and/or pattern of their spikes (Proposition 3.5). This distinguishes the walks-with-jumps model from models in which similarity between spike trains is determined on the basis of detailed firing patterns.

The point-of-no-return property provides a natural substrate for decision-making based on dynamics, as it indicates that sufficient differences in the initial portion of spike trains will force their endpoints to lie in non-overlapping sets. In this sense, it is similar to the notion that behavioral decisions might correspond to neural activity patterns falling into alternative basins of attraction (Wang 2012) or integration of a decision variable to a threshold (Shadlen and Newsome 2001). However, the subdivision of spike trains in the walk-with-jumps model has a distinctive aspect: by Theorem 1.8, as the spike trains within each subset evolve further, they will split again (and again) into subsets with separate fates. This provides a natural way to model decisions or classifications that are refined as time progresses – or, more broadly, coarse-to-fine processing (Hegde 2008).

Related to the point-of-no-return behavior is the phenomenon that the initial portion of a spike train has a disproportionate effect on its endpoint. Moreover, even the insertion of a segment of time without a spike at the beginning of a spike train will influence its endpoint. Both of these characteristics are biologically plausible. Many neurons are more sensitive to transients than to steady inputs (e.g. Chance et al. (1998)). As a result, the meaning of a spike train as interpreted by the recipient neuron will be disproportionately dependent on the initial spikes.

In the walks-with-jumps model, inserting “empty” time, i.e., changing the latency of a response, will change its endpoint. For this to have a biological correlate, it is a prerequisite that the brain has access to a “start time.” While a purely passive sensory system may not provide such a reference, coupling sensation to a motor act—e.g., a whisk, a sniff, or a saccade—builds in a reference point. Moreover, in many sensory systems (including somatosensation (Ahissar and Kleinfeld 2000; Kleinfeld et al. 2006), olfaction (Shusterman et al. 2011; Smear et al. 2011; Warner et al. 2024), and vision (Boi et al. 2017), the impact of incoming sensory information depends on its timing relative to this reference. Once a start time has been established, changes in latency suffice to make fine spatial judgments (Kleinfeld et al. 2006), to distinguish odors, to signal contrast (Reich et al. 2001), and to disambiguate contrast from other spatial information (Gawne et al. 1996). Of note, neural response latency can also be influenced by the prior history of visual stimulation (Bair et al. 2002). While at first glance this finding suggests that latency shifts should not have perceptual consequences, it is important to recognize that perception, also, depends on the dynamics of visual inputs (Rucci et al. 2025).

Another way in which the walk-with-jumps model differs from other measures of spike train similarity relates to the superposition property. The superposition property states that for two spike trains A and B, the distance between them unchanged when the same set of spikes are added to both, i.e., that D(A,B)=D(A+X,B+X), where “+” denotes superposition of spike trains. The superposition property holds for a spike count distance, as well as for distances based on firing rate envelope and the “spike metric” (Victor and Purpura 1997), but it does not hold for walks with jumps. For example, if A and B are two distinct spike trains that initially diverge but then converge to the same endpoint (guaranteed by Proposition 3.5), adding a single spike after their point of divergence will result in separating their endpoints.

The superposition property, however, is neither “good” nor “bad,” but rather, has implications for domains in which the walk-with-jumps model is likely to be applicable. For example, in the representation of a domain such as color (a three-parameter domain in which, approximately, the level of activity of neurons correspond to projections onto the axes (Derrington et al. 1984; Zaidi et al. 2013)), superposition is appropriate, as superposition of stimuli, approximately, leads to addition of neural responses. But in other domains, such as olfaction, this is decidedly not the case (Inagaki et al. 2020).

More generally, we emphasize that we are not proposing that walks-with-jumps replace existing models, merely that its properties make it worth considering in specific contexts—and we suggest some empirical tests in Section 6.1. Such contexts include those in which decisions or classifications are made, especially if such decisions or classifications are refined over time, in which the domain being represented has a hyperbolic similarity structure (Zhou et al. 2018), or in which the superposition property may not be appropriate. On the other hand, for representation of perceptual spaces that are Euclidean or approximately so, the walk-with-jumps model has no evident advantages.

Finally, we note that the family of walks-with-jumps models form a parameterized continuum: one end of the continuum (walk speed or jump angle set to 0) yields a model in which spike counts are simply accumulated over time. Increasing the value of these parameters results in a progressively increasing prominence of the model’s distinctive characteristics. When these parameters are large enough, the endpoints of walks with jumps distinguish the spike trains that generate them at a fine level of detail, as even small shifts in spike timing will trigger the “point of no return” property. In between, a mixture of properties emerge: a point of no return for spike trains that are sufficiently different, coexisting with the existence of distinct firing patterns that lead to the same endpoints.

We now turn to developing a formal structure that supports the above claims.

### Formal definitions and main results

As is standard, we idealize the activity of a single neuron during a time interval as a sample drawn from a point process, i.e., a sequence of stereotyped electrophysiological events (action potentials) which together constitute a “spike train". Formally:

#### Definition 1.1

Let $T\in \mathbb {R}^+$ and let $k\in \mathbb {N}$. A **spike train (over the period [0,T]) with** *k* **spikes** is a sequence $t_1 \le t_2 \le t_3 \le t_4 \le \ldots \le t_k$ with each $t_i\in [0,T]$. We say that **its spikes occur** at the times $t_i$.

That is, a neuron producing the above spike train has spikes at the times $t_i$ and is quiescent over the periods from $t_i$ to $t_{i+1}$ for each $0\le i\le k-1$. In this standard model, the information represented by the neuron over the time interval [0, *T*] is fully contained in its set of spike times.

**Fig. 1:**
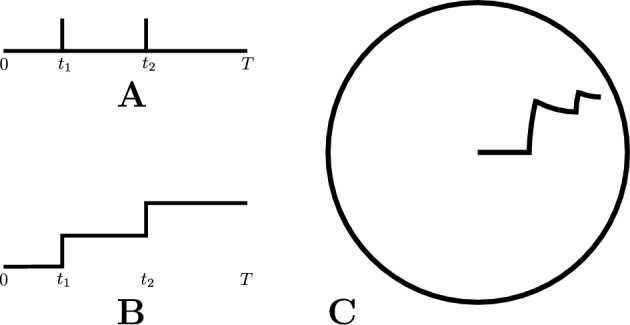
Three different ways of visualizing a spike train

Figure 1A is a standard visual representation of a spike train over the period [0, *T*] with spikes at $t_1< t_2 < T$. An equivalent conceptualization is shown in Figure 1B: here, activity is represented by the counting process *F*(*t*), where $F(t) = \int _0^t f(s)\,\textit{ds}$ and *f*(*t*) is a sequence of (unit-mass) delta-functions corresponding to the point process schematized in Figure 1A.

This second conceptualization has another interpretation: the neuron’s activity is motion of a point along a path in $\mathbb {R}^2$, in which the point moves in one direction at constant speed when there is no spike, and then abruptly jumps in another (orthogonal) direction at the times that a spike occurs. That is, Figure 1B conceptualizes a neuron’s activity as a “walk with jumps” in $\mathbb {R}^2$, with jumps of length 1 at right angles to the walk direction.

Figure 1C shows a walk with jumps determined by the same data, but in the *hyperbolic plane* $\mathbb {H}^2$ not $\mathbb {R}^2$. (Here and below, $\mathbb {H}^2$ always refers to the unique complete Riemannian two-manifold having constant sectional curvature $-1$, but see Remark 1.3.) Such walks with jumps in $\mathbb {H}^2$ are the main object of study in this paper. We regard the walk itself as capturing the neuron’s activity over time, and the walk’s endpoint as an invariant of this activity. Formally:

#### Definition 1.2

A **walk with jumps model** is specified by choosing an *origin* $o\in \mathbb {H}^2$ and unit tangent vector $\textbf{v}$ at *o*, and the following *parameters*:

- a *walk speed* $s>0$, *jump angle* $\theta \in (-\pi ,\pi )$, and *jump length* $\ell >0$.

We call the tuple $(o,\textbf{v},s,\theta ,\ell )$ the **data of the model**. Within any walk with jumps model, a **walk with jumps** is specified by a *duration* $T>0$ and *jump data*:

- a sequence $(t_1,\hdots ,t_n)$ of *jump times* ($n\ge 0$), where $0 \le t_1<\hdots < t_n \le T$, and a *burst vector* $(j_1,\hdots ,j_n)$ with natural number entries, taken to be $(1,\hdots ,1)$ if not otherwise specified.

The walk’s *number of jumps* is $N = \sum _{i=1}^n j_i$; we also call it a **walk with** *N* **jumps**.

#### Remark 1.3

In Definition 1.2, changing the origin *o* and/or the unit tangent vector $\textbf{v}$ does not change any qualitative feature of the model, as these changes result in models that are equivalent via an isometry of $\mathbb {H}^2$. On the other hand, changing model parameters may change qualitative features. In particular, scaling the walk speed and jump length simultaneously by a uniform parameter is equivalent to rescaling the curvature.

Within a walk with jumps model, the data of a walk with jumps encode the following set of instructions for movement:

Beginning at *o*, walk along the geodesic ray in the direction of $\textbf{v}$ at speed *s* until time $t_1$. At time $t_1$, instantly jump a distance of $\ell \cdot j_1$ along an axis through the current location at a counterclockwise angle of $\theta$ to the original; then proceed at speed *s* along a geodesic ray at angle $-\theta$ to the jump axis. Continue until time $t_2$; jump a distance $\ell \cdot j_2$ along an axis at angle $\theta$ to the current one; then proceed right again along an axis at angle $-\theta$ to the jump axis, etc.

Having executed the instructions above and halted at time *T*, our avatar’s final location is the **endpoint** of the walk with jumps. It carries the initial tangent vector $\textbf{v}$ with it by parallel transport, finally yielding a tangent vector at the endpoint which we call the **endvector**.

#### Remark 1.4

Some neurons exhibit *bursts*: sequences of spikes in very short succession. In the limit of a vanishingly small interspike interval, these multiple jumps approach the behavior of a single jump whose magnitude is a natural number multiple of the stereotypical single spike. We capture this behavior in Definition 1.2, with a “burst vector" rather than allowing consecutive jump times to be equal. While these two different approaches are mathematically equivalent (i.e., they produce walks with identical behaviors), using burst vectors will be technically convenient when we write walks with jumps as words in isometries of $\mathbb {H}^2$ specified by the data *o*, ${\textbf {v}}$, *s*, $\theta$, $\ell$ and *T*, see Section 2.1. Note though that this equivalence is purely a mathematical one; we are not claiming that a burst in which spike times are separated is biologically equivalent to a large-magnitude spike, or that spikes within a burst are equivalent to each other. Indeed, for nonzero separations, the non-commutativity of isometries of $\mathbb {H}^2$ means that this is not the case.

Ultimately, we are interested in comparing the characteristics of the walks with jumps model to findings in the experimental literature. Here however we focus on establishing basic facts about our construction using classical techniques of hyperbolic geometry and geometric group theory.

The central question of this paper is of the extent to which the jump data of a walk with jumps can be recovered from its endpoint *p*, or the finer information $(p,\textbf{w})$, where $\textbf{w}\in T_p\mathbb {H}^2$ is the endvector, for a fixed set of initial data. We offer a spectrum of partial answers to this question in Sections 2, 3 and 4, in both positive and negative directions, reflecting the truism that hyperbolic space is “Euclidean at small scales and treelike at large scales”. What this should mean for walks with jumps is fleshed out in Section 1.3. We show there that for the analog of a walk with jumps construction in the Euclidean plane, the endpoint is determined solely by the duration and number of jumps. In contrast, for the analogous construction in a tree, any two distinct jump *patterns* produce distinct endpoints.

Section 2 develops calculus tools for understanding how perturbations of a walk’s jump times affect its endpoint-endvector pair. In Section 2.1 we prove Lemma 2.7, a basic fact describing how the data of a walk with jumps uniquely prescribes an isometry carrying its origin-initial vector pair $(o,\textbf{v})$ to $(p,\textbf{w})$. Using this, in Corollary 2.14 we define a differentiable map $\psi _k$ whose domain is a simplex $T_k$ encoding all walks with *k* jumps in a given model with a fixed duration, and whose target space is the unit tangent bundle of $\mathbb {H}^2$. The map $\psi _k$ takes $\textbf{s}\in T_k$ to the endpoint-endvector pair $(p,\textbf{w})$ of the walk with jumps corresponding to $\textbf{s}$. The main technical result of Section 2, Proposition 2.17, describes the regularity and derivative of $\psi _k$.

#### Proposition 2.17

Fix $k\in \mathbb {N}$ at least 3, the data $(o,{\textbf {v}},s,\theta ,\ell )$ of a walk with jumps model, and a duration $T>0$, and let $T_k = \{(s_1,\hdots ,s_k)\,|\, 0< s_1< \cdots< s_k< T\}\subset \mathbb {R}^k$ as in Definition 2.9. The map $\psi _k: T_k\rightarrow \textit{UT}\mathbb {H}^2$ from Corollary 2.14 that takes $\textbf{s}\in T_k$ to the endpoint-endvector pair of the corresponding walk with jumps is $C^1$, and its derivative has full rank on a dense open subset $U\subset T_k$.

The continuous differentiability of $\psi _k$ demonstrates that small perturbations to a spike train do not *much* affect the endpoint-endvector pair of the corresponding walk with jumps. In fact, Corollary 2.20 asserts that for $k>3$, if a walk’s sequence of jump times lies in the open set *U* of the Proposition, then there exist arbitrarily small perturbations of this sequence that do not change the endpoint-endvector pair at all. On the other hand, by Corollary 2.21, for $k\ge 3$ the set of endpoints of walks with jumps is the closure of its interior in $\mathbb {H}^2$—in particular, there is an *open* subset of the unit tangent bundle of $\mathbb {H}^2$ consisting of endpoint-endvector pairs.

In Section 3 we move beyond perturbations, constructing walks with significantly different jump patterns that still share an endpoint-endvector pair. Proposition 3.5 describes such pairs belonging to the same walk with jumps model, and sharing a number of jumps and duration, but with arbitrarily large gaps between jump times of the first and the second. This construction does rely, however, on there being not too large a gap between the first jump times of the two walks with jumps. In Section 5 we give conditions that eliminate this possibility.

But first, in Section 4 we establish some basic structure results by applying the tools of hyperbolic geometry to a class of paths associated to walks with jumps.

#### Definition 1.5

For a walk with jumps with the data of Definition 1.2, the associated **walk-with-jumps path** is the broken geodesic in $\mathbb {H}^2$ obtained by joining the origin *o* to the pre-jump location $p_1$ at time $t_1$; joining $p_1$ to the post-jump location $q_1$ at time $t_1$; then joining $q_1$ to the pre-jump location $p_2$ at time $t_2$ and so on. Its *i*th **walk segment** joins $q_{i-1}$ to $p_i$ (or *o* to $p_i$, if $i=1$, or $q_n$ to the endpoint, if $i = n+1$), and its *i*th **jump segment** joins $p_i$ to $q_i$. We parametrize it continuously on $\left[ 0,sT + \ell \cdot \sum _{i=1}^n j_i\right]$, mapping 0 to *o*, by parametrizing each geodesic segment in turn by arclength.

A quick inductive argument shows that the pre- and post-jump locations $p_i$ and $q_i$ at time $t_i$ occur at parameter values $st_i + \ell \sum _{k=1}^{i-1} j_k$ and $st_i + \ell \sum _{k=1}^i j_k$, respectively, for $i\in \{1,\hdots ,n\}$.

#### Remark 1.6

The endpoints of the walk-with-jumps path associated to a walk with jumps are the origin *o* and the endpoint of the walk with jumps itself, as defined above. Moreover, its endvector is the outward-pointing unit tangent vector to the final segment of the walk-with-jumps path or, if the final jump time $t_n$ equals *T*, the unit tangent vector at an angle of $-\theta$ from that vector.

In Section 4.1 we lay the groundwork for our further results by associating two collections of pairwise disjoint geodesics, the *walk* and *jump axes*, to each walk with jumps. The main result of Section 4.2, Proposition 4.11 asserts that any walk-with-jumps path is a *quasigeodesic*, meaning the hyperbolic distance between any two points on it is well-approximated by their distance along the path (see Definition 4.7). This implies that walks in the same model with the same duration but different enough numbers of jumps have different endpoints, see Corollary 4.13.

The main result of Section 5 shows that a pair of conditions, which we now define, ensures that distinct walks with jumps have different endpoints.

#### Definition 1.7

For $\epsilon > 0$, two walks with jumps in the same walk with jumps model that share a duration $T>0$ are *distinct to resolution* $\epsilon$ if their sequences of jump times $(s_1,\hdots ,s_m)$ and $(t_1,\hdots ,t_n)$ satisfy:

1. for each $i\le \min \{m,n\}$, either $s_i = t_i$ or $|s_i-t_i|>\epsilon$; and
2. there exists some *i* such that $s_i\ne t_i$, or $m\ne n$.

For $R_{min}>0$, a walk with jumps has *minimum refractory length* $R_{min}$ if its burst vector is $(1,\hdots ,1)$—ie. it has no bursts—and for $i\ne j$, $|t_i-t_j|\ge R_{min}$.

While these conditions may not appear geometrically natural, they are meaningful in biological contexts in which it is not possible to discern differences at arbitrarily small resolutions, and in which physical constraints prevent a neuron from firing again too soon after it has fired once. At suitable scales, they prohibit distinct walks with jumps from having the same endpoint.

#### Theorem 1.8

Given $R_{\min }>0$, the parameters $(s,\theta =\pi /2,\ell )$ of a walk with jumps model, and a duration $T>0$, let

$$\begin{aligned} \epsilon = \frac{1}{s}\cosh ^{-1}\left( \frac{\cosh (sR_{min})+1}{\cosh (sR_{min})-\tanh ^2\ell } \right) . \end{aligned}$$

Any two walks with jumps in the given model, having duration *T* and minimum refractory length $R_{min}$, that are distinct to resolution $\epsilon$, have distinct endpoints.

Note that for any fixed positive *s* and $\ell$, $\epsilon$ defined as above can be made arbitrarily small by choosing $R_{\min }$ large enough. We prove a stronger version of Theorem 1.8 in Section 5 as Theorem 5.4. This more precise version also yields Corollary 5.5, on the existence of trees whose edges are traversed by walk-with-jumps paths.

Section 6 lists further questions, including some that are biologically motivated and others motivated by the hyperbolic-geometric phenomenon called *quasigeodesic stability*, see Proposition 6.1.

### Comparing models of a single neuron’s activity

In this section we compare and contrast qualitative features of “walk with jumps“ style constructions in various metric contexts, beginning below with that of $\mathbb {R}^2$. As we will show, for trajectories in $\mathbb {R}^2$, the endpoint is determined solely by the number of spikes and the time interval. That is, two spike trains with the same endpoint can have very different firing patterns. However, in $\mathbb {H}^2$, the arrangement of the spikes also influences the endpoint – a feature that is appealing from the point of view that in neural circuits, spike timing, as well as spike count, conveys information. Our overall focus is on the relationship between the endpoint and the path, for example, to what extent do spike trains with the same endpoint necessarily have similar paths?

#### Example 1.9

Consider a walk with jumps model as in Definition 1.2, but in $\mathbb {R}^2$ not $\mathbb {H}^2$, with origin (0, 0), unit tangent vector (1, 0), walk speed 1, jump angle $\pi /2$, jump length 1. For a walk with jumps having duration *T* and jump times $0\le t_1 \le t_2 \le \cdots \le t_k \le T$, the corresponding walks with jumps path has endpoint:

$$\begin{aligned} (T,k) = (t_1,0) + \sum _{i=1}^{k}\left( (t_{i+1}-t_i),1\right) + (T-t_k,0) \end{aligned}$$

That is, the path’s endpoint is determined entirely by the walk’s duration and number of spikes.

In Example 1.9, the walk path endpoints do not take into account the timing of individual spikes in a spike train. In contrast, spike timing does affect path endpoints for walks with jumps in $\mathbb {H}^2$. The result below uses hyperbolic trigonometry to quantify the effect produced by changing the timing of a single spike, when the jump angle is $\pi /2$. In fact we show that if two such walks have a large enough gap in the timing of their first spikes, then they can never have the same endpoint.

#### Proposition 1.10

Consider two walks with jumps in the same model (in $\mathbb {H}^2$), having parameters $(s,\theta = \pi /2$,$\ell )$. If each walk has a single jump, and this occurs at time 0 in the first and at time *T* in the second, then the distance *d* between the two walks’ endpoints satisfies:

$$\begin{aligned} \cosh (d)-1 = (\cosh (\ell )-1)(\cosh (sT)-1)(\cosh (\ell )\cosh (sT)-1). \end{aligned}$$

Regardless of how many jumps each has, if the first walk has its first jump at time 0 and the second has its first jump at time $t_1$ satisfying

$$\begin{aligned} \sinh (\ell )\sinh (s\, t_1)\ge 1, \end{aligned}$$

then the two walks have distinct endpoints.

**Fig. 2:**
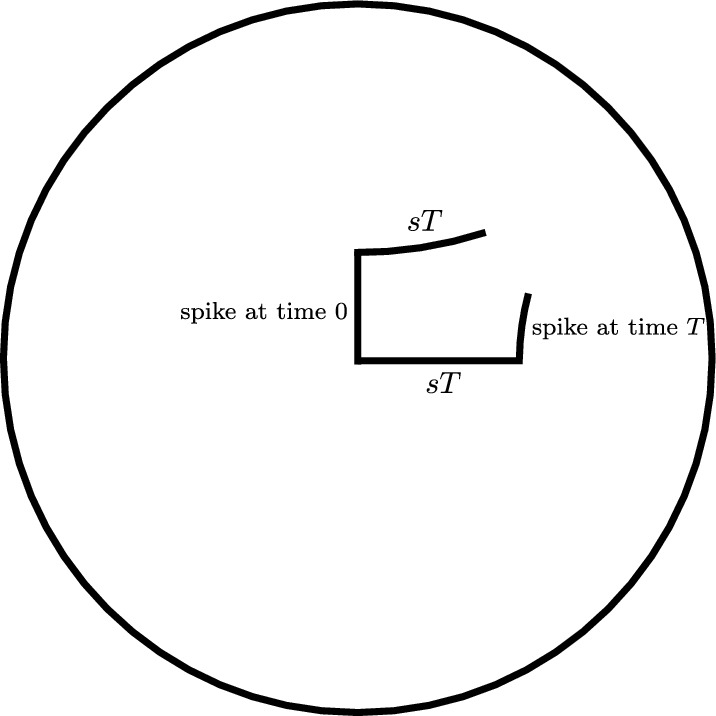
This figure shows two hyperbolic walks with jumps over the same time period, *T*,  with a single jump. In one walk, the jump occurs at the beginning of the time period, and in the other it occurs at the end

#### Proof

The union of walks-with-jumps paths (in the sense of Definition 1.5) for the first two walks considered here (having one jump each) is pictured in Figure 2, using the Poincaré disk model with *o* at the origin. The vertical line segment in the Figure is the first walk’s jump segment, which has length $\ell$ in $\mathbb {H}^2$. The horizontal line segment is the second walk’s walk segment, of length *sT*. The other two segments are each contained in circular arcs meeting the unit circle perpendicularly—the form of any hyperbolic geodesic not containing the origin in the Poincaré disk model.

Let $q_1$ be the far endpoint from *o* of the first walk’s jump segment, at the top left corner of the shape made by the walk-with-jumps paths of Figure 2, and let $p_1'$ be the far endpoint from *o* of the second walk’s walk segment. The shortest arc joining the first walk’s endpoint to the hyperbolic geodesic containing the second walk’s walk segment meets that segment at an angle of $\pi /2$ at a point $p_0'$, and therefore defines a *Lambert quadrilateral* *Q*—one with three right angles—whose vertices are *o*, $q_1$, $p_0'$, and the first walk’s endpoint.

The first walk’s jump segment is one side of *Q*, of length $\ell$, and its walk segment is another, of length *sT*. Let *x* be the length of the side of *Q* joining *o* to $p_0'$, *y* the distance from the first walk’s endpoint to $p_0'$, and $\gamma$ the interior angle at the first walk’s endpoint. A hyperbolic law of cosines for Lambert quadrilaterals gives:

1 $$\begin{aligned} \sinh (\ell )\sinh (x) = \cos (\gamma ). \end{aligned}$$

See Theorem 3.5.9 of (Ratcliffe 2019). A law of cosines for more general quadrilaterals having right angles at two adjacent vertices, recorded as (Ratcliffe (2019), Th. 3.5.8), specializes in the case of *Q* to give $\cosh (sT) \sin (\gamma ) = \cosh (x)$. Using the first equation to replace $\sin (\gamma )$ in the second, and solving for $\sinh (x)$, yields the formula below:

$$\begin{aligned} \sinh (x) = \frac{\sinh (sT)}{\sqrt{\cosh ^2(sT)\sinh ^2(\ell )+1}}. \end{aligned}$$

This implies in particular that $x<sT$. Section VI.3.3 of Fenchel (1989) gives several more trigonometric formulas for these more general quadrilaterals. One with the form of a hyperbolic law of sines specializes to $\sinh (y) = \cosh (sT)\sinh (\ell )$ for *Q*. We apply a final formula from Fenchel (1989) to another such quadrilateral, this one with vertices at the first and second walks’ endpoints, and at $p_0'$ and $p_1'$. It gives:

$$\begin{aligned} \cosh (d) = -\sinh (y)\sinh (\ell ) + \cosh (y)\cosh (\ell )\cosh (sT-x). \end{aligned}$$

The Proposition’s first conclusion is obtained by substituting for *x* and *y* in this formula using those obtained above, and simplifying.

We now consider the second situation contemplated in the Proposition, in which both walks may have many jumps but the first walk still has its first jump at time 0. As in the previous case, let $q_1$ be the far endpoint from *o* of the first walk’s jump segment, and let $p_1'$ be the far endpoint of the second walk’s first walk segment. The distance from *o* to $q_1'$ is again $\ell$, and in this case from *o* to $p_1'$ is $st_1$.

The geodesic ray from $q_1$ that contains the first walk’s next walk segment is at a right angle to its first jump segment; and likewise, the geodesic ray containing the second walk’s first jump segment is at right angles to its first walk segment. If these rays intersect, then their subsegments joining $q_1$ and $p_1'$ to the point of intersection form a Lambert quadrilateral, together with the first walk’s first jump segment and the second walk’s first walk segment. If so then by the law of cosines for Lambert quadrilaterals, as in (1) above, we would have $\sinh (\ell )\sinh (s t_1) = \cos (\gamma )$, where $\gamma$ is the angle at which the rays meet.

Thus if $\sinh (\ell )\sinh (s t_1)\ge 1$ then the two geodesic rays do not intersect. In this case we can deduce that the walks will not share endpoints, since the first walk’s endpoint is on the other side from *o* of the geodesic containing the ray from $q_1$, and the second walk’s endpoint is on the other side from *o* of the geodesic containing the ray from $p_1'$ . (We will prove this in Lemma 4.5 below.) $\square$

**Fig. 3:**
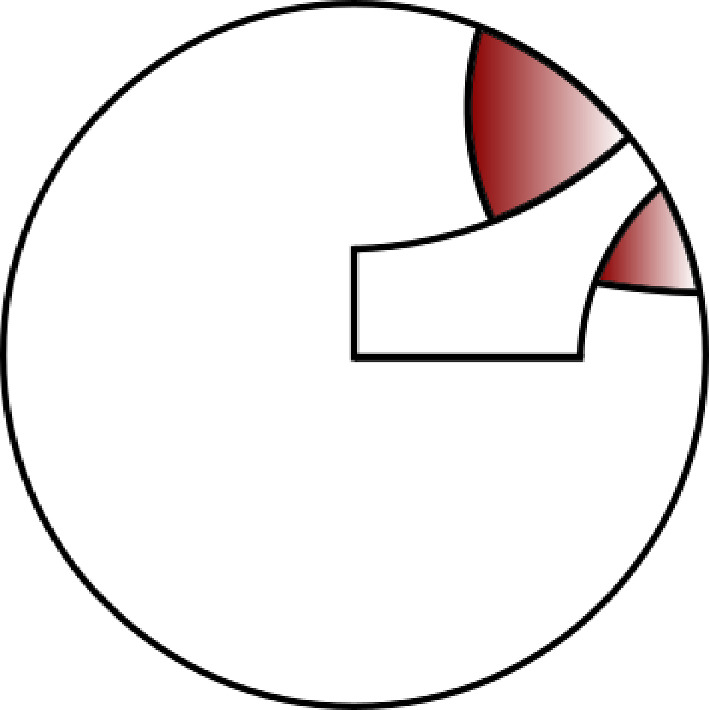
Disjoint future quadrants

The train of thought that finishes off the proof above motivates the notion of “future quadrant”, an intersection of half-planes associated to an initial segment of a walk with jumps that is guaranteed to contain the walk’s endpoint. This is formally defined in Definition 4.6. See Figure 3 for the picture relevant to the proof of Proposition 1.10.

The behavior described in Proposition 1.10 is reminiscent of paths in a tree, which, once they diverge, can never come back together. Another motivating example for the walk with jumps construction is a strategy for encoding a(n approximation to a) spike train by a walk in a tree, as we now describe.

Given a spike train *S* over a time period *t*, we can encode a discrete approximation to *S* in a binary sequence *w* of length *n* as follows: for each $1\le i \le n$, if *S* has a spike in $[\left( \frac{i-1}{n}\right) t, \left( \frac{i}{n}\right) t]$, then *i*th entry of *w* is 1. If there is no spike in $[\left( \frac{i-1}{n}\right) t, \left( \frac{i}{n}\right) t]$, then the *i*th entry of *w* is 0. We can view these discrete approximations of spike trains geometrically by embedding them in a binary tree:

#### Example 1.11

Let $\mathcal {T}$ be a binary tree with root $p_0$. A binary sequence of length *n*, $w = w_1w_2\ldots w_n$ where $w_i\in \{0,1\}$, encodes a path of length *n* from $p_0$ to a vertex of $\mathcal {T}$ as follows: at the root proceed left if $w_1=1$ and right if $w_1=0$. At the $(i-1)$th vertex of the path, proceed left if $w_i=1$ or right if $w_i=0$.

If $\gamma ,\sigma$ are paths (encoded as binary sequences) of length *m*, *n* respectively, then the distance between their endpoints can be computed as follows: if $\gamma = \gamma _1\gamma _2\ldots \gamma _m$ and $\sigma = \sigma _1\sigma _2\ldots \sigma _n$, let *d* be the length of the maximal common prefix of $\gamma$ and $\sigma$. Then the distance between the endpoints of $\sigma$ and $\gamma$ is equal to $(m-d)+(n-d)$.

Walks-with-jumps paths in $\mathbb {H}^2$ have geometry similar to the paths encoded by binary sequences in Example 1.11 with some distinct advantages. First, walks-with-jumps paths can model continuous processes. In the tree *T*, the binary sequences

$$\begin{aligned} LRLLLLLLLLLLLLLL \qquad \text { and } \qquad LLLLLLLLLLLLLLLL \end{aligned}$$

are quite similar, but their endpoints will be far apart. The walks-with-jumps model has the feature that non-identical paths that can track each other closely if the event times are similar– behavior that is biologically appealing, since the underlying biophysics of neuronal activity necessarily places a limit on the precision of spike times. This feature more effectively accounts for noise that appears in data.

## Distinct walks with identical endpoints

In the final section we will produce a fairly general set of conditions in which two walks with jumps are guaranteed to have distinct endpoints, suggesting that to some extent, the endpoint “encodes” the times of the jumps. But first, here, we show that this encoding is not fully faithful. In Section 2.2 we show this via a general but non-constructive argument that implies coincident endpoints for walks with jumps whose jump times are not very different; following that, in Section 3, we create explicit examples of walks with jumps that have distinct jump times but reach the same endpoint, and the jump times of these walks can differ substantially.

### Background and set-up

Standard facts about the geometry of $\mathbb {H}^2$ imply the following:

#### Lemma 2.1

Given the data $\left( o\in \mathbb {H}^2,\textbf{v}\in \textit{UT}_o\mathbb {H}^2,s>0, \theta \in (-\pi ,\pi ),\ell >0\right)$ of a walk with jumps model as in Definition 1.2, there is a unique hyperbolic isometry $f\in \operatorname {PSL}_2(\mathbb {R})$ and one-parameter family $(g_t)_{t\in \mathbb {R}}\subset \operatorname {PSL}_2(\mathbb {R})$ satisfying:

- The $g_t$ share an axis $\gamma _g$ through *o* in the direction of $\textbf{v}$, and for each *t*, $g_t$ translates *o* a distance of *st* along $\gamma _g$.
- The axis $\lambda _f$ of *f* contains *o*, and *f* translates the origin *o* a distance of $\ell$ along $\lambda _f$ at an angle of $\theta$ counterclockwise from $\gamma _g$.

#### Remark 2.2

Some remarks about Lemma 2.1:

- The data of the walk with jumps model is recovered from *f* and the $g_t$ as follows: $o = \lambda _f\cap \gamma _g$, $\left. \frac{d}{dt}\right| _{t=0}g_t(o) = s\textbf{v}$, $\ell = d(o,f(o))$, and $\theta$ is the counterclockwise angle from $\textbf{v}$ to the direction vector from *o* to *f*(*o*).
- By replacing *f* and $(g_t)$ with isometries of $\mathbb {H}^2$ that do not satisfy all requirements of Lemma 2.1, we can produce “walks with jumps” satisfying different specifications — for instance with travel along horocyclic arcs or geodesic equidistants.

#### Definition 2.3

Let *w* be a word on $\{f,g_t:\,t\in \mathbb {R}^{\ge 0}\}$. The **syllable length** of *w* is the smallest number $m\ge 1$ so that *w* can be grouped as

2 $$\begin{aligned} w = g_{s_1}f^{n_2}g_{s_3} f^{n_4}\ldots f^{n_{m-1}}g_{s_m}. \end{aligned}$$

where $n_i\in \mathbb {Z}^{>0}$ and $s_i> 0$ for $1<i<m$. We call each of the $f^{n_i},g_{s_i}$ **syllables** of *w*. We say *w* is written in **syllabic form** if expressed as in (2).

We will henceforth abuse notation and use a word *w* in syllabic form to refer to both the word itself and the isometry that it represents.

#### Remark 2.4

The syllabic form of a word should always have an odd number of syllables where the first and last syllables are $g_{s_0}$ and $g_{s_m}$ (which may be the identity). The intermediate syllables cannot be the identity.

#### Example 2.5

The syllable length of $w_1 = g_6 f^2 g_2g_3 f^3f$ is 5 because we rewrite $w_1$ in syllabic form as $g_6f^2g_{5} f^4g_0$. The first syllable of the syllabic form is $g_6$, the second syllable is $f^2$ and the third syllable is $g_5$.

#### Definition 2.6

Let $(o,\,\textbf{v},\,s,\,\theta ,\,\ell )$ be the data of a walk with jumps model. For a walk with jumps in the model having duration *T* and jump data $(t_1,\hdots ,t_n)$, and $(j_1,j_2,\hdots ,j_n)$, taking *f* and $\{g_t\}_{t\in \mathbb {R}}$ as in Lemma 2.1, let

$$\begin{aligned} w = g_{t_1}f^{j_1}g_{t_2-t_1}f^{j_2}\cdots g_{t_{n}-t_{n-1}}f^{j_n}g_{T-t_{n}}. \end{aligned}$$

Let $w_0$ be the identity, $w_1 = g_{t_1}$ and for all $1\le i\le |w|$, let $w_i$ be the product of the first *i* syllables of *w*. The *walk axes* are the geodesics $\gamma _i \doteq w_{2(i-1)}(\gamma _g)$ for $i\in \{1,\hdots ,n+1\}$, and the *jump axes* are the geodesics $\lambda _i \doteq w_{2i-1}(\lambda _f)$ for $i\in \{1,\hdots ,n\}$, where $\gamma _g$, $\lambda _f$ are as in Lemma 2.1.

#### Lemma 2.7

For a walk with jumps in the model with data $(o,\,\textbf{v},\,s,\,\theta ,\,\ell )$, having duration *T* and jump data $(t_1,\hdots ,t_n)$, and $(j_1,j_2,\hdots ,j_n)$, the endpoint-endvector pair is $(w(o),w_*(\textbf{v}))$, where *w* from Definition 2.6 is regarded as an isometry of $\mathbb {H}^2$, and $w_*$ is its derivative at *o*. Moreover, for each $i\in \{1,\hdots ,n+1\}$, $\gamma _i$ from Definition 2.6 contains the *i*th walk segment of the associated walk-with-jumps path, as described in Definition 1.5, and $\lambda _i$ contains the *i*th jump segment for $i\le n$.

#### Proof

The proof proceeds by induction on *n*. In all cases let $\textbf{u}$ be the unit vector at *o* tangent to $\lambda _f$ pointing in the direction of *f*(*o*), so that the counterclockwise angle from $\textbf{v}$ to $\textbf{u}$ is $\theta$.

Assume $n=1$, and suppose first that $t_1 = T$. Then the associated walks-with-jumps path consists of two segments: the first, of length $st_1$, contained in $\gamma _g$—which by definition equals $\gamma _1$—with one endpoint at *o* and $\textbf{v}$ pointing into it; the second, of length $j_1\ell$, sharing the other endpoint $p_1$ of the first and at a counterclockwise angle of $\theta$ from its outward-pointing tangent vector. The derivative of $g_{t_1}$ at *o* takes $\textbf{v}$ to this outward-pointing vector, since $g_{t_1}$ translates a distance of $st_1$ along $\gamma _g$, so it also takes $\textbf{u}$ to the vector pointing into the second segment at *p*. Therefore the second segment is contained in $g_{t_1}(\lambda _f) = \lambda _1$.

In this case $w = g_{t_1}f^{j_1}g_0$ in syllabic form (where $g_0$ is the identity). Note that $f^{j_1}(o)$ lies on $\lambda _f$ at a distance of $j_1\ell$ from *o* in the direction of $\textbf{u}$. Therefore $g_{t_1}$ carries the segment of $\lambda _f$ bounded by *o* and $f^{j_1}(o)$ to the second segment of the walk-with-jumps path, so $g_{t_1}f^{j_1}(o)$ is the path’s endpoint. Similarly, the derivative of $f^{j_1}$ at *o* carries $\textbf{v}$ to a vector at $f^{j_1}(o)$ at a clockwise angle of $\theta$ from the outward-pointing unit vector to the segment of $\lambda _f$ bounded by *o* and $f^{j_1}(o)$; it then follows from the chain rule that $w_*(\textbf{v})$ is the walk’s endvector.

Still taking $n=1$, now suppose that $t_1 < T$. Then the associated walks-with-jumps path has three segments: the first two as in the previous case; and the third, of length $s(T-t_1)$, sharing the endpoint $q_1\ne p_1$ of the second segment and at a *clockwise* angle of $\theta$ from its outward-pointing tangent vector. Thus by the previous case, the inward-pointing tangent vector to the third segment is $d(g_{t_1}f^{j_1})_o(\textbf{v})$, and it follows that this segment lies in $\gamma _2\doteq g_{t_1}f^{j_1}(\gamma _g)$. Moreover since $g_{T-t_1}(o)$ is the point on $\gamma _g$ at distance $s(T-t_1)$ from *o* in the direction of $\textbf{v}$, $(g_{t_1}f^{j_1})(g_{T-t_1}(o))$ is the far endpoint of the third walk segment from $q_1$.

Since $w = g_{t_1}f^{j_1}g_{T-t_1}$ in this case, the above states exactly that *w*(*o*) is the endpoint of the walk with jumps. And since $d(g_{T-t_1})_o(\textbf{v})$ is the outward-pointing tangent vector at $g_{T-t_1}(o)$ to the sub-arc of $\gamma _g$ bounded by *o* and $g_{T-t_1}(o)$, it follows from the chain rule as in the previous subcase that $w_*(\textbf{v})$ is the endvector of the walk with jumps. This proves the Lemma’s $n=1$ case.

The $n>1$ case is analogous, after applying the inductive hypothesis to the sub-walk with jumps having the same initial data, duration $t_n$, jump times $(t_1,\hdots ,t_{n-1})$, and burst vector $(j_1,\hdots ,j_{n-1})$. The product $w_{2n-1}$ of the first $2n-1$ syllables of *w* then plays the role of $g_{t_1}$ in the $n=1$ case above, as by the inductive hypothesis, $(w_{2n-1}(o),(w_{2n-1})_*(\textbf{v}))$ is the endpoint-endvector pair of the sub-walk with jumps. That is, $w_{2n-1}(o)$ is the far endpoint from *o* of the walk-with-jumps path associated to the sub-walk—ie. the union of the first $2n-1$ segments of the path associated to the walk specified in the Lemma—and $(w_{2n-1})_*(\textbf{v})$ is the outward-pointing tangent vector to the $(2n-1)^{\textrm{st}}$ segment. The two sub-cases now follow as in the the $n=1$ case, replacing $j_1$ there with $j_n$ here and $T-t_1$ with $T-t_n$. $\square$

### Perturbing jump times without changing the endpoint

We begin with a standard result that combines two assertions: that the isometry group of $\mathbb {H}^2$ acts transitively on it, and that an isometry is determined by where it sends a single point and tangent vector at that point. Below $\textit{UT}\,\mathbb {H}^2$ is the unit tangent bundle of $\mathbb {H}^2$, consisting of pairs $(p,\textbf{v})$ for $p\in \mathbb {H}^2$ and $\textbf{v}$ a unit-length tangent vector at *p*, and $\operatorname {SL}_2(\mathbb {R})$ is the group of $2\times 2$ real matrices with determinant 1.

#### Proposition 2.8

For a fixed $o\in \mathbb {H}^2$ and unit tangent vector $\textbf{v}$ at *o*, there is a diffeomorphism $\operatorname {PSL}_2(\mathbb {R})\rightarrow \textit{UT}\,\mathbb {H}^2$ that sends $w\in \operatorname {PSL}_2(\mathbb {R})$ to the pair $(w(o),w_*(\textbf{v}))$ in $\textit{UT}\,\mathbb {H}^2$, for $\operatorname {PSL}_2(\mathbb {R}) = \operatorname {SL}_2(\mathbb {R})/\{\pm I\}$.

#### Proof

Taking $\mathbb {H}^2$ as the set of $z\in \mathbb {C}$ with positive imaginary part, $\operatorname {SL}_2(\mathbb {R})$ acts on it via Möbius transformations: for $z\in \mathbb {H}^2$ and $a,b,c,d\in \mathbb {R}$ such that $ad-bc = 1$,

$$\begin{aligned} \begin{pmatrix} a & b \\ c & d \end{pmatrix}.z = \frac{az+b}{cz+d}. \end{aligned}$$

This action extends to a smooth $\operatorname {SL}_2(\mathbb {R})$-action on the tangent bundle $\mathbb {H}^2\times \mathbb {C}$ by taking $$\left( {\begin{smallmatrix} a & b \\ c & d \end{smallmatrix}}\right) $$ to act on the tangent vector $v\in \mathbb {C}$ at $z\in \mathbb {H}^2$ by multiplication by its derivative $1/(cz+d)^2$. This action preserves the hyperbolic Riemannian metric and hence restricts to an action on

$$\begin{aligned} \textit{UT}\mathbb {H}^2 = \{ (z,v) \in \mathbb {H}^2\times \mathbb {C}\,|\, |v| = \Im z \} \end{aligned}$$

It is an exercise to check that the $\operatorname {SL}_2(\mathbb {R})$-action on $\mathbb {H}^2$ is transitive, and that the stabilizer of *i* is

$$\begin{aligned} \operatorname {SO}(2) = \left\{ \begin{pmatrix} \cos \theta & -\sin \theta \\ \sin \theta & \cos \theta \end{pmatrix} \right\} . \end{aligned}$$

Such a matrix acts on a tangent vector *v* at *i* by multiplication by $1/(i\sin \theta + \cos \theta )^2 = e^{-2i\theta }$, i.e. as rotation by $-2\theta$. Therefore the extended action on $\textit{UT}\mathbb {H}^2$ is also transitive, i.e.,  $\textit{UT}\mathbb {H}^2$ is a *homogeneous space* of $\operatorname {SL}_2(\mathbb {R})$. Furthermore for any unit complex number *v*, regarded as a tangent vector to $\mathbb {H}^2$ at *i*, the stabilizer in $\operatorname {SL}_2(\mathbb {R})$ of (*i*, *v*) is the set

$$\begin{aligned} \left\{ \begin{pmatrix} \cos (n\pi ) & -\sin (n\pi ) \\ \sin (n\pi ) & \cos (n\pi )\end{pmatrix}\right\} = \{ \pm I \}. \end{aligned}$$

Proposition 2.8 now follows directly from the characterization of homogeneous spaces, see e.g. (Lee (2013), Theorem 21.18). This asserts that for a Lie group *G* acting on a homogeneous space *X*, and any fixed $x_0\in X$, the smooth map $G\rightarrow X$ given by $g\mapsto g.x_0$ induces a diffeomorphism $G/H\rightarrow X$, where *H* is the stabilizer of $x_0$ in *G*. $\square$

#### Definition 2.9

For $T\in \mathbb {R}^+$ and $k\in \mathbb {N}$, let

$$\begin{aligned} T_k:=\{(s_1,\ldots ,s_k):\, 0< s_1<s_2<s_3<\ldots< s_k < T\}\subset \mathbb {R}^k \end{aligned}$$

and

$$\begin{aligned} \overline{T}_k:=\{(s_1,\ldots ,s_k):\, 0 \le s_1\le s_2\le s_3\le \ldots \le s_k \le T\}\subset \mathbb {R}^k. \end{aligned}$$

#### Remark 2.10

$\overline{T}_k$ is a simplex in $\mathbb {R}^k$ which we view as encoding all possible walks with *k* jumps and duration *T* in a given model. A tuple $\textbf{s} = (s_1,\hdots ,s_k)$ in $\overline{T}_k$ encodes the same jump data as the pair $(t_1,\hdots ,t_n)$, $(j_1,\hdots ,j_n)$ from Definition 1.2, where $n\le k$ is the number of distinct $s_i$ and for each $l\le n$, $t_l$ is a distinct such $s_i$, and $j_l$ is the number of $s_i$ equal to $t_l$.

$T_k$ is the interior of $\overline{T}_k$, an open subset of $\mathbb {R}^k$ consisting of paths that have burst vector $(1,1,\hdots ,1)$.

#### Lemma 2.11

Given the data $(o,{\textbf {v}},s,\theta ,\ell )$ of a walk with jumps model as in Definition 1.2, and $k\in \mathbb {N}$, $T>0$: choose matrices representing the isometries *f* and $(g_t)_{t\in \mathbb {R}}$ of Lemma 2.1 so that *f* has positive trace and $g_0 = I$. Continuing to refer to these matrices as *f* and $g_t$, define $\Omega : \mathbb {R}^k \rightarrow \operatorname {SL}_2(\mathbb {R})$ by

$$\begin{aligned} \Omega (\textbf{s}) = g_{s_1}f g_{s_2-s_1}f\cdots g_{s_{k}-s_{k-1}}fg_{T-s_{k}}, \end{aligned}$$

for $\textbf{s} = (s_1,\hdots ,s_k)$. This map is continuously differentiable.

#### Remark 2.12

If $\textbf{s}\in \overline{T}_k$, for $\overline{T}_k$ from Definition 2.9, then a computation shows that $\Omega (\textbf{s})\in \operatorname {SL}_2(\mathbb {R})$ represents the isometry *w* from Definition 2.6.

#### Proof

We will show that $\Omega$ has continuous partial derivatives, from which continuous differentiability will follow. To compute its *i*th partial derivative at $\textbf{s} = (s_1,\hdots ,s_k)\in T_k$, we write $w\doteq \Omega (\textbf{s})$ as $L_i\,f\,R_i$, for

3 $$\begin{aligned} L_i = g_{s_1}fg_{s_2-s_1}\cdots f g_{s_i-s_{i-1}}\quad {and}\quad R_i = g_{s_{i+1}-s_i}f\cdots fg_{T-s_k}. \end{aligned}$$

For small $t>0$, we then have

$$\begin{aligned} \Omega (s_1,\hdots ,s_{i-1},s_i+t,s_{i+1},\hdots ,s_k) = L_i\,g_tfg_{-t}\, R_i \end{aligned}$$

We may thus compute $\frac{\partial }{\partial s_i}\Omega (\textbf{s}) = \left. \frac{d}{dt}\right| _{t=0}(D_i\circ C)(t)$ with the chain rule, where $D_i: \operatorname {SL}_2(\mathbb {R})\rightarrow \operatorname {SL}_2(\mathbb {R})$ is given by $D_i(X) = L_i\,X\,R_i$ and $C(t) = g_{t}\, f\, g_{-t}$.

We compute $C'(0)$ using the “product rule for matrix multiplication”: for differentiable families $(A_t)$ and $(B_t)$ of $2\times 2$ matrices,

$$\begin{aligned} \left. \frac{d}{dt}\right| _{t=0}(A_tB_t) = A_0\left[ \left. \frac{d}{dt}\right| _{t=0} B_t\right] + \left[ \left. \frac{d}{dt}\right| _{t=0} A_t\right] B_0. \end{aligned}$$

This yields $C'(0) = \mathfrak {g}f - f\mathfrak {g}$, where $\mathfrak {g}= \left. \frac{d}{dt}\right| _{t=0}g_t\in \mathfrak {sl}_2(\mathbb {R})$, since $g_0 = I$. Since the action of $D_i$ on $\operatorname {SL}_2(\mathbb {R})$ restricts its action as a linear map of $M_2(\mathbb {R})$, the vector space of $2\times 2$ matrices, $D_i$ is its own derivative and we have

$$\begin{aligned} \frac{\partial }{\partial s_i}\Omega (\textbf{s}) = L_i(\mathfrak {g}f - f\mathfrak {g})R_i. \end{aligned}$$

The only $\textbf{s}$-dependence above lies in the left- and right-multipliers $L_i$ and $R_i$. These vary continuously with $\textbf{s}$, by repeated applications of the continuity of the multiplication map on $\operatorname {SL}_2(\mathbb {R})$, and for the same reason so does $\partial \Omega /\partial s_i$. $\square$

#### Remark 2.13

The partial derivatives computed above belong to the tangent space of $\operatorname {SL}_2(\mathbb {R})$ at *w*. Translating each back to the identity using left-multiplication by $w^{-1}$ yields $R_i^{-1}f^{-1}(\mathfrak {g}f - f\mathfrak {g})R_i = R_i^{-1}\left( f^{-1}\mathfrak {g}f - \mathfrak {g}\right) R_i$.

$$ f^{-1}(\mathfrak {g}f - f\mathfrak {g}) = 2s\sin \theta \sinh \ell \begin{pmatrix} -\cos \theta \sinh \ell & \cosh \ell +\sin \theta \sinh \ell \\ \cosh \ell + \sin \theta \sinh \ell & \cos \theta \sinh \ell \end{pmatrix} $$

#### Corollary 2.14

For the data $o,{\textbf {v}},s,\theta ,\ell$ of a walk with jumps model, $k\in \mathbb {N}$, and $T>0$, the map $\psi _k: T_k\rightarrow \textit{UT}\mathbb {H}^2$ given by composing the diffeomorphism from Proposition 2.8 with $\Omega$ from Lemma 2.11 is continuously differentiable.

#### Remark 2.15

The map $\psi _k$ defined above takes a sequence of jump times $\textbf{s}\in T_k$ to the endpoint-endvector pair of the walk with jumps determined by the prescribed initial data, $\textbf{s}$, and duration *T*. In particular, the image of $\psi _k$ is the collection of endpoints of all walks with *k* jumps, duration *T*, and initial data specified above.

#### Proposition 2.16

For $k>3$ and $T_k\subset \mathbb {R}^k$ as in Definition 2.9, given the data $(o,{\textbf {v}},s,\theta ,\ell )$ of any walk with jumps model and $T>0$, the map $\psi _k:T_k\rightarrow \textit{UT}\mathbb {H}^2$ defined in Corollary 2.14 is not injective on any open subset $U\subseteq T_k$. That is: for any such *U* there exist distinct walks with jumps in the given model, each with *k* jumps, duration *T*, and whose sequence of jump times is contained in *U*, having the same endpoint-endvector pair.

#### Proof

Since $\textit{UT}\mathbb {H}^2$ is a 3-manifold we may assume that $\psi _k|_U$ maps to $\mathbb {R}^3$, by replacing *U* with the smaller open set $U\cap \psi _k^{-1}(V)$ for a chart open set $V\subset \textit{UT}\mathbb {H}^2$ intersecting $\psi _k(U)$, and post-composing with the chart map. If $\psi _k|_U$ were injective then by Brouwer’s Invariance of Domain Theorem, $\psi |_U$ would be a homeomorphism between *U* and $\psi (U)\subset \mathbb {R}^3$. However this contradicts the fact, a standard exercise in algebraic topology (cf. (Hatcher (2002), Th. 2.26)), that no open subset of $\mathbb {R}^k$ is homeomorphic to an open subset of $\mathbb {R}^3$ when $k>3$. $\square$

Proposition 2.16 uses only the continuity of the map $\psi _k$ to show that it is non-injective *near every point of* $T_k$, *at arbitrarily small scales*. Interpreted in terms of walks with jumps, this implies that for any given walk with at least three jumps, one can find distinct others which each differ from the original – hence also from each other – by an arbitrarily small perturbation of jump times, and have the same endpoint-endvector pair as each other (but not necessarily as the original walk with jumps). The next result gives a calculus tool for obtaining still finer information.

#### Proposition 2.17

Fix $k\in \mathbb {N}$ at least 3, the data $(o,{\textbf {v}},s,\theta ,\ell )$ of a walk with jumps model, and a duration $T>0$, and let $T_k = \{(s_1,\hdots ,s_k)\,|\, 0< s_1< \cdots< s_k< T\}\subset \mathbb {R}^k$ as in Definition 2.9. The map $\psi _k: T_k\rightarrow \textit{UT}\mathbb {H}^2$ from Corollary 2.14 that takes $\textbf{s}\in T_k$ to the endpoint-endvector pair of the corresponding walk with jumps is $C^1$, and its derivative has full rank on a dense open subset $U\subset T_k$.

#### Proof

Using the upper half-plane model we may assume without loss of generality that $o = i$ and $\textbf{v} = (1,0)$. In this case the isometries $(g_t)_{t\in \mathbb {R}}$ and *f* of Lemma 2.1 are represented by matrices

$$ g_t = \begin{pmatrix} \cosh (st) & \sinh (st) \\ \sinh (st) & \cosh (st) \end{pmatrix}\ {and}\ f = \begin{pmatrix} \cosh \ell + \sin \theta \sinh \ell & \cos \theta \sinh \ell \\ \cos \theta \sinh \ell & \cosh \ell - \sin \theta \sinh \ell \end{pmatrix} $$

Note that $g_0$ is the identity and *f* has positive trace, as required in Lemma 2.11. For $\mathfrak {g} = \left. \frac{d}{dt}\right| _{t=0}g_t\in \mathfrak {sl}_2(\mathbb {R})$, a computation now yields that $$\mathfrak {g} = s\left( {\begin{smallmatrix} 0 & 1 \\ 1 & 0 \end{smallmatrix}}\right) $$, and hence that

$$\begin{aligned} \mathfrak {v}\doteq \mathfrak {g}f - f\mathfrak {g} = 2s\sin \theta \sinh \ell \begin{pmatrix} 0 & -1 \\ 1 & 0 \end{pmatrix}. \end{aligned}$$

This matrix features in the description of $\frac{\partial }{\partial s_i}\Omega (\textbf{s})$ in the proof of Lemma 2.11.

#### Claim 2.17.1

For any $\textbf{s}= (s_1,\hdots , s_k)\in \overline{T}_k$, and any *i*, $\frac{\partial }{\partial s_i}\Omega (\textbf{s})\ne \textbf{0}$.

#### Proof of Claim

Appealing to Remark 2.13, we find that the claim holds if and only if $f^{-1}\mathfrak {v}\ne \textbf{0}$. This in turn can be easily verified using the descriptions of *f* and of $\mathfrak {v}$ recorded immediately above, together with the hypotheses on *s*, $\theta$ and $\ell$ implying that all scale factors of $\mathfrak {v}$ are positive. $\square$

#### Claim 2.17.2

For any $\textbf{s}= (s_1,\hdots , s_k)\in \overline{T}_k$, and any $i < k$, $\frac{\partial }{\partial s_i}\Omega (\textbf{s})$ and $\frac{\partial }{\partial s_{i+1}}\Omega (\textbf{s})$ are linearly independent.

#### Proof of claim

These two matrices are linearly *dependent* if and only if one is a scalar multiple of the other; ie, using the proof of Lemma 2.11, if and only if

$$\begin{aligned} k\, L_i\,\mathfrak {v}\,R_i = L_{i+1}\,\mathfrak {v}\,R_{i+1}\quad \Leftrightarrow \quad k\,\mathfrak {v} (R_iR_{i+1}^{-1}) = (L_i^{-1}L_{i+1})\mathfrak {v} \end{aligned}$$

for some $k\in \mathbb {R}$, where $L_i$ and $R_i$ are as described in (3). Using this description, the equality above is equivalent to the one below, defining $\delta _i = s_{i+1} - s_i$:

$$\begin{aligned} k\,\mathfrak {v}\, (g_{\delta _i}f) = (g_{\delta _i}f)\,\mathfrak {v}. \end{aligned}$$

If we write $$g_{\delta _i}f= \left( {\begin{smallmatrix} a & b \\ c & d \end{smallmatrix}}\right) $$ then since $\mathfrak {v}$ is a scalar multiple of $$\left( {\begin{smallmatrix} 0 & -1 \\ 1 & 0\end{smallmatrix}}\right) $$, the above equality is equivalent to following equations simultaneously holding:

$$\begin{aligned} -kc = b,\quad kd = a,\quad ka=d,\quad -kb = c. \end{aligned}$$

Noting that we have $ad-bc = 1$, it follows that if the above all hold then $k = 1$, $a = d$, and $b = -c$. Performing the computations, we find that

$$\begin{aligned} a = d \quad \Leftrightarrow \quad \sin \theta \cosh (s\delta _i)\sinh \ell = 0 \end{aligned}$$

But this does not hold for $\theta \in (0,\pi )$ and $\ell >0$, regardless of whether $\delta _i = 0$. $\square$

#### Claim 2.17.3

For any fixed *i*, $1< i < k$, there is an open dense set of $\textbf{s}\in \overline{T}_k$ such that $\frac{\partial }{\partial s_{i-1}}\Omega (\textbf{s})$, $\frac{\partial }{\partial s_i}\Omega (\textbf{s})$, and $\frac{\partial }{\partial s_{i+1}}\Omega (\textbf{s})$ are linearly independent.

#### Proof of Claim

For arbitrary $\textbf{s}= (s_1,\hdots , s_k)\in \overline{T}_k$, taking $L_i$ and $R_i$ (and $L_{i\pm 1}$, $R_{i\pm 1}$) as in (3), the proof of Lemma 2.11 gives

$$ \frac{\partial }{\partial s_{i-1}}\Omega (\textbf{s}) = L_{i-1}\mathfrak {v} R_{i-1},\quad \frac{\partial }{\partial s_{i}}\Omega (\textbf{s}) = L_{i}\mathfrak {v} R_{i},\ \ {and}\ \ \frac{\partial }{\partial s_{i+1}}\Omega (\textbf{s}) = L_{i+1}\mathfrak {v} R_{i+1}.$$

Multiplying the entire collection on the left by $L_{i}^{-1}$ and on the right by $R_{i}^{-1}$, we find that the linear independence of this collection is equivalent to that of:

$$\begin{aligned} \mathcal {B}_0 \doteq \left\{ \ (L_{i}^{-1}L_{i-1})\,\mathfrak {v}\, R_{i-1}R_{i}^{-1},\ \ \mathfrak {v},\ \ (L_{i}^{-1}L_{i+1})\,\mathfrak {v}\,(R_{i+1}R_{i}^{-1}) \ \right\} . \end{aligned}$$

Taking $\delta _i = s_{i+1}-s_i$ and $\delta _{i-1} = s_i - s_{i-1}$, we further have:

$$ L_{i-1}^{-1}L_i = g_{\delta _{i-1}} f = R_{i-1}R_i^{-1},\ {and}\ L_{i}^{-1}L_{i+1} = g_{\delta _i} f = R_{i}R_{i+1}^{-1}. $$

Writing $g_{\delta _{i-1}}f$ as $A_{i-1}$ and $g_{\delta _i}f$ as $A_i$, the collection in question has the form:

$$ \mathcal {B}_0 = \left\{ \ A_{i-1}^{-1}\mathfrak {v}\,A_{i-1},\ \mathfrak {v},\ A_i\,\mathfrak {v}\,A_{i}^{-1} \right\} $$

Taking $$A_{i-1} = \left( {\begin{smallmatrix} a & b \\ c & d \end{smallmatrix}}\right) $$ and $$A_i =\left( {\begin{smallmatrix} x & y \\ z & w \end{smallmatrix}}\right) $$, and dividing out the common scale factor $2s\sin \theta \sinh \ell >0$ yields:

$$\begin{aligned} \mathcal {B} = \left\{ \begin{pmatrix} -ab-cd & -b^2-d^2 \\ a^2+c^2 & ab+cd \end{pmatrix}, \begin{pmatrix} 0 & -1 \\ 1 & 0 \end{pmatrix}, \begin{pmatrix} xz+yw & -x^2-y^2 \\ z^2+w^2 & -xz-yw \end{pmatrix} \right\} . \end{aligned}$$

If there existed $\alpha ,\beta ,\gamma \in \mathbb {R}$ such that the linear combination

$$\begin{aligned} \alpha \begin{pmatrix} -ab-cd & -b^2-d^2 \\ a^2+c^2 & ab+cd \end{pmatrix} + \beta \begin{pmatrix} 0 & -1 \\ 1 & 0 \end{pmatrix} + \gamma \begin{pmatrix} xz+yw & -x^2-y^2 \\ z^2+w^2 & -xz-yw \end{pmatrix} = \begin{pmatrix} 0 & 0 \\ 0 & 0 \end{pmatrix}, \end{aligned}$$

then by considering upper-left entries we find that

$$\begin{aligned} \alpha (ab+cd) = \gamma (xz+yw), \end{aligned}$$

and by considering lower-left and upper-right entries that

$$\begin{aligned} \alpha (a^2+c^2) + \gamma (z^2+w^2) = -\beta = \alpha (b^2+d^2) + \gamma (x^2+y^2). \end{aligned}$$

Eliminating variables we thus find that either $\alpha = \beta = \gamma = 0$ or

4 $$\begin{aligned} \frac{xz+yw}{x^2+y^2-z^2-w^2} = \frac{ab+cd}{a^2-b^2+c^2-d^2} \end{aligned}$$

The collection $\mathcal {B}$ is thus linearly *dependent* if and only if equation (4) holds. Note that its left-hand side depends only on $\delta _i = s_{i+1}-s_i$, whereas the right depends only on $\delta _{i-1} = s_i-s_{i-1}$, among quantities that vary with $\textbf{s}$. In particular, $\frac{\partial }{\partial s_{i+1}} a = \frac{\partial }{\partial s_{i+1}} b = \frac{\partial }{\partial s_{i+1}} c = \frac{\partial }{\partial s_{i+1}} d = 0$. We claim that the corresponding partial derivative of the left-hand side is positive.

This follows from direct computation. Taking $S_i = \sinh (s\delta _i)$ and $C_i = \cosh (s\delta _i)$, $S_\ell = \sinh \ell$ and $C_{\ell } = \cosh \ell$, and $s_\theta = \sin \theta$ and $c_\theta = \cos \theta$, we have:

$$\begin{aligned} A_i&= \begin{pmatrix} x & y \\ z & w \end{pmatrix} = \begin{pmatrix} C_iC_\ell + s_\theta C_i S_\ell + c_\theta S_i S_\ell & S_i C_\ell - s_\theta S_i S_\ell + c_\theta C_i S_\ell \\ S_i C_\ell + s_\theta S_i S_\ell + c_\theta C_i S_\ell & C_iC_\ell - s_\theta C_i S_\ell + c_\theta S_i S_\ell \end{pmatrix} \end{aligned}$$

Further computation now yields

$$\begin{aligned} xz+yw&= 2S_iC_i(S_{\ell }^2+C_{\ell }^2) + 2c_{\theta }(S_i^2+C_i^2)S_{\ell }C_{\ell } \\&= \sinh (2s\delta _i)C_{2\ell } + c_{\theta }\cosh (2s\delta _i)S_{2\ell } \\ x^2 + y^2 - z^2 - w^2&= 4s_\theta S_\ell C_\ell = 2s_{\theta }S_{2\ell }, \end{aligned}$$

where $C_{2\ell } = \cosh (2\ell )$ and $S_{2\ell } = \sinh (2\ell )$. The hypotheses $\theta \in (0,\pi /2]$ ensures that $c_{\theta }\ge 0$, and $S_i>0$ since $\textbf{s}\in T_k$. Thus using that $\delta _i = s_{i+1}-s_i$ and the computation above, we find that $\frac{\partial }{\partial s_{i+1}}(xz+yw) > 0$. Again by the computation above, the denominator $x^2+y^2-z^2-w^2$ of the left side of (4) does not depend on $\delta _i$; hence the left-hand side of (4) increases with $s_{i+1}$ as claimed.

But this further implies Claim 2.17.3, since at any $\textbf{s}= (s_1,\hdots ,s_k)\in T_k$ where equation (4) holds, increasing $s_{i+1}$ while holding $s_i$ and $s_{i-1}$ constant produces points of $T_k$ arbitrarily close to $\textbf{s}$ where (4) does not hold. Therefore the set of $\textbf{s}$ where $\frac{\partial }{\partial s_{i-1}}\Omega (\textbf{s})$, $\frac{\partial }{\partial s_i}\Omega (\textbf{s})$, and $\frac{\partial }{\partial s_{i+1}}\Omega (\textbf{s})$ are linearly independent is dense. And it is also open, being the complement of the solution set to (4). $\square$

Given that *U* contains the open dense set described in Claim 2.17.3 for any fixed *i*, the claim immediately implies the result. $\square$

The next example shows that the set *U* of Proposition 2.17 where the derivative of $\psi _k$ has full rank may be a proper subset of $T_k$.

#### Example 2.18

Let us now consider a walk with jumps satisfying the hypotheses of Proposition 2.17, specialize to the case that $\theta = \pi /2$, and consider the equation (4) in this case. The computations below that equation specialize to:

$$\begin{aligned} xz + yw&= \sinh (2s\delta _i)\cosh (2\ell ) \\ x^2+y^2-z^2-w^2&= 2\sinh (2\ell ) \\ ab+cd&= \sinh (2s\delta _{i-1}) \\ a^2-b^2+c^2-d^2&= 2\cosh (2s\delta _{i-1})\sinh (2\ell ) \end{aligned}$$

Setting the appropriate products equal to each other as in (4), and rearranging terms, we thus obtain the following equation:

$$\begin{aligned} \sinh (2s\delta _i) = \frac{\tanh (s\delta _{i-1})}{\cosh (2\ell )}. \end{aligned}$$

This equation is satisfied if and only if $\frac{\partial }{\partial s_{i+1}}\Omega (\textbf{s})$ lies in the span of $\frac{\partial }{\partial s_{i-1}}\Omega (\textbf{s})$ and $\frac{\partial }{\partial s_i}\Omega (\textbf{s})$.

We may regard it as recursively prescribing the value of $\delta _i$, given $\delta _{i-1}$. A given value of $\delta _1$ therefore uniquely determines the set of values $\delta _i$, $i>1$, such that the span of all $\frac{\partial }{\partial s_i}\Omega (\textbf{s})$ is two-dimensional.

#### Remark 2.19

We could ask whether two walks with different durations can ever have the same endpoint. This may be less biologically relevant.

We close this subsection by recording some consequences of the technical Proposition 2.17 for endpoints and endvectors of walks with jumps.

#### Corollary 2.20

Fix $k\in \mathbb {N}$ greater than 3, the data $(o,{\textbf {v}},s,\theta , \ell )$ of a walk with jumps model, and a duration $T>0$, and let $T_k\subset \mathbb {R}^k$ parametrize the set of duration-*T* walks with *k* jumps in the given model as in Definition 2.9. For any such walk with jumps whose sequence of jump times lies in the dense open set $U\subset T_k$ of Proposition 2.17, there exist arbitrarily small perturbations of its sequence of jump times yielding walks with jumps sharing its endpoint and endvector.

#### Proof

Recall that *U* is defined to be the set of $\textbf{s}\in T_k$ at which the derivative of the map $\psi _k: T_k\rightarrow \textit{UT}\mathbb {H}^2$ from Corollary 2.14 has full rank, meaning rank three since $\textit{UT}(\mathbb {H}^2)$ is three-dimensional. Since $\psi _k$ is continuously differentiable, it is a standard consequence of the Implicit Function Theorem that for any $\textbf{s}\in U$, the level set of $\psi _k$ containing $\textbf{s}$ intersects *U* in a submanifold of dimension $k-3$. Recalling the interpretation of $\psi _k$ from Remark 2.15, we see that the small perturbations of the present Corollary’s statement lie on this submanifold, which has positive dimension since by hypothesis $k>3$. $\square$

#### Corollary 2.21

For any fixed $k\ge 3 \in \mathbb {N}$, data $(o,{\textbf {v}},s, \theta ,\ell )$ of a walk with jumps model, and any duration $T>0$, the set of endpoints of walks with jumps in the given model that have *k* jumps and duration *T* is the closure of its interior. In particular, it has non-empty interior.

#### Proof

The set of endpoints of walks with jumps is the image of the simplex $T_k$ of Definition 2.9 under the composition of the bundle projection $\textit{UT}\mathbb {H}^2\rightarrow \mathbb {H}^2$ with the map $\psi _k: T_k\rightarrow \textit{UT}\mathbb {H}^2$ from Corollary 2.14. The bundle projection is a submersion, so by Proposition 2.17 this composition has full rank derivative at every point of the open dense subset $U\subset T_k$ identified there. As in the previous proof, the implicit function theorem now implies that for every $\textbf{s}\in U$, there is an open neighborhood of $\psi _k(\textbf{s})$ contained in $\psi _k(U)\subset \psi _k(T_k)$. Thus $\psi _k(U)$ is contained in the interior of the set of walk endpoints. Moreover, since *U* is dense in $T_k$, its image is dense in the set of endpoints. $\square$

#### Remark 2.22

The diameter of the set of endpoints of walks with jumps in a given model having $\theta =\pi /2$ and other data identical to that of Corollary 2.21, meaning the supremum of the pairwise distances between these endpoints, can be explicitly bounded below along the lines of Proposition 1.10. A computation parallel to the proof of that result shows that the distance *d* between the endpoints of the walks with jumps corresponding to the vertices $(0,\hdots ,0)$ and $(T,\hdots ,T)$ of $T_k$ satisfies:

$$\begin{aligned} \cosh (d)-1 = (\cosh (k\ell )-1)(\cosh (sT)-1)(\cosh (k\ell )\cosh (sT)-1). \end{aligned}$$

The diameter of the set of endpoints is thus bounded below by *d*, which can be seen to increase linearly with any of the individual quantities featured in its definition.

This exhibits a stark qualitative difference between the Euclidean and hyperbolic contexts: if one replaced “$\mathbb {H}^2$” by “$\mathbb {R}^2$” in its statement then the resulting set of endpoints would have diameter 0, being a single point.

#### Proposition 2.23

Given the data $(o,{\textbf {v}},s,\theta ,\ell )$ of a walk with jumps model, and $T>0$, there exist two distinct sequences of jump times $(t_1,\ldots ,t_n)$ and $(t_1',\ldots ,t_n')$ that the corresponding walks with jumps in the model, both of duration *T*, have the same endpoints. In particular, the associated isometries are equal:

$$\begin{aligned} w = g_{t_1}fg_{t_2-t_1}f\cdots g_{t_{n}-t_{n-1}}fg_{T-t_{n}}\ { and }\ w' = g_{t_1'}fg_{t_2'-t_1'}f\cdots g_{t_{n}'-t_{n-1}'}fg_{T-t_{n}'}. \end{aligned}$$

#### Proof

By Proposition 2.16, the map $\psi$ is not injective. Then $(w(o),w_*({\textbf {v}})) = (w'(o),w_*'({\textbf {v}}))$. By Proposition 2.8, $w = w'$. $\square$

#### Definition 2.24

A **semigroup** is a set together with an associative operation. A **free semigroup** is one that is isomorphic to the semigroup generated by a set with no relations.

#### Corollary 2.25

Given the data, $(o,{\textbf {v}},s,\theta ,\ell )$ and $T>0$, the isometry *f* and the one parameter family $g_t$ do not generate a free semigroup.

## More different walks with identical endpoints

The previous section’s results, notably Corollary 2.20, show that in any walk with jumps model there are distinct walks with the same duration and number of jumps, having identical endpoints. It is the nature of that approach to produce walks whose jump times are small perturbations of each other. In this section we will show by example that much more significant variation in jump times can still yield walks with jumps that have identical endpoints. We begin with Proposition 3.2, showing that a walk with a single, immediate jump can be well approximated for an arbitrary time by one in which a burst of two jumps occurs after a lag, followed by a regular sequence of jumps. We will then use a couple of tricks to turn a pair of such walks into a pair with identical durations and endpoints, in Proposition 3.5.

The family of examples produced here do not represent the full spectrum of possible ways in which walks with “significantly different” jump patterns may have identical endpoints; indeed, we do not feel that we have a firm handle on this issue at the moment. The construction we present makes non-generic choices at certain points for the sake of convenience. For instance, “two jumps” could easily be replaced by “*n* jumps” in the paragraph above, for arbitrary $n\ge 2$. And we fix the jump angle at $\pi /2$ immediately below in order to take advantage of certain helpful hyperbolic trigonometric formulas for right-angled hyperbolic polygons.

In the lemma below, a *pentagon* is a concatenation of distinct geodesic arcs $\alpha _1,\hdots ,\alpha _5$—its *sides*—such that $\alpha _i$ and $\alpha _{i+1}$ share an endpoint for each $i<5$, as do $\alpha _1$ and $\alpha _5$. Its *vertices* are $v_i \doteq \alpha _i\cap \alpha _{i+1}$, for $i<5$, and $v_5 \doteq \alpha _5\cap \alpha _1$. Its *angle* at $v_i$ ($i=1$ to $i=4$) is measured counterclockwise from $\alpha _{i+1}$ to $\alpha _i$, or for $i=5$, from $\alpha _1$ to $\alpha _5$. A pentagon is *self-intersecting* if non-adjacent sides intersect.

### Lemma 3.1

For any $\ell >0$ there exist $0< X_0 < X_1 \doteq \cosh ^{-1}(\coth \ell \tanh (2\ell ))$, such that for any $x\in (X_0,X_1)$ there is a self-intersecting pentagon $\alpha _1,\hdots ,\alpha _5$ satisfying the following conditions:

- $\alpha _1$ has length $\ell$, $\alpha _2$ length *x*, and $\alpha _3$ length $2\ell$;
- the angles at $v_5$, $v_1$, $v_2$ and $v_3$ all equal $\pi /2$; and
- $$\alpha _3$$ intersects $$\alpha _5$$.

The angle $\delta$ at $v_4$ satisfies $\displaystyle { \lim _{x\rightarrow X_0^+} \delta = 0}$, and $\displaystyle {\lim _{x\rightarrow X_1^-} \delta = \frac{\pi }{2} - \sin ^{-1}\left( \frac{\cosh \ell }{\cosh (2\ell )}\right) }$.

**Fig. 4:**
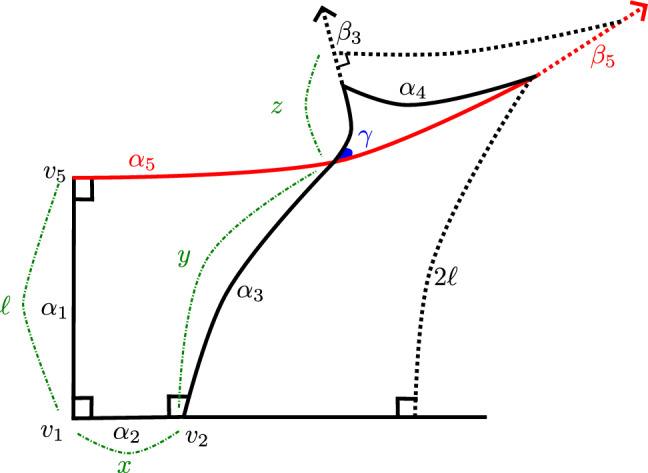
The self-intersecting pentagon from Lemma 3.1

Figure 4 depicts the pentagon from Lemma 3.1, along with ancillary quantities from its proof.

### Proof

A quadrilateral in $\mathbb {H}^2$ with three right angles (called a “Lambert quadrilateral” in the classical geometry literature), is determined by the lengths of its two sides that have right angles at both of their vertices. If these two side lengths are $\ell$ and *x* then the final vertex angle $\gamma < \pi /2$ satisfies:

5 $$\begin{aligned} \cos \gamma = \sinh \ell \sinh x.\quad \mathrm{(See (Ratcliffe, 2019, Thrm. 3.5.9).)} \end{aligned}$$

Note that this implies in particular that the product $\sinh \ell \sinh x$ can be at most 1. If this product is at least 1 then the two geodesics meeting the sides with lengths $\ell$ and *x* at right angles to their endpoints do not meet in $\mathbb {H}^2$; if it equals 1 then the two sides are “parallel”, ie. asymptotic, the quadrilateral has finite area, and we say it has a single “ideal vertex”.

If $\sinh \ell \sinh x < 1$ then applying (Ratcliffe (2019), Thrm. 3.5.8) to the Lambert quadrilateral above gives the relation between $\ell$, $\gamma$, and the length *y* of the side opposite the one with length $\ell$ recorded in the first equality below. Equation (5) then allows us to replace $\sin \gamma$ in the denominator, yielding the second equality:

$$\begin{aligned} \cosh y = \frac{\cosh \ell }{\sin \gamma } = \frac{\cosh \ell }{\sqrt{1-\sinh ^2 x\sinh ^2\ell }}. \end{aligned}$$

Note that if we fix $\ell$ and allow *x* to vary, this formula defines *y* as an *increasing* function of *x*. Setting $y = 2\ell$ and solving for *x* yields the formula for $X_1$ given in the Lemma’s statement.

By the above, for any $x<X_1$, the following construction yields a geodesic arc $\alpha _3$ crossing a geodesic ray $\beta _5$ that contains $\alpha _5$: Let $\alpha _1$ and $\alpha _2$ be arcs of lengths $\ell$ and *x*, respectively, meeting at right angles at a point $v_1$; let $\alpha _3$ be a geodesic arc of length $2\ell$ meeting $\alpha _2$ at right angles at its endpoint $v_2$ opposite $v_1$, so that $\alpha _1$ and $\alpha _3$ belong to the same half-space bounded by the geodesic containing $\alpha _2$; and let $\beta _5$ be the geodesic ray meeting $\alpha _1$ at right angles at its endpoint $v_5$ opposite $v_1$, and contained in the same half-space bounded by the geodesic containing $\alpha _1$ as $\alpha _3$.

Still taking $x<X_1$, for $\alpha _3$ and $\beta _5$ as above, let $c = \alpha _3\cap \beta _5$ and let $\beta _3$ be the geodesic ray with endpoint *c*, contained in the geodesic containing $\alpha _3$, in the opposite half-space bounded by the geodesic containing $\beta _5$ from $\alpha _2$. There exists a point on $\beta _3$ at a distance $z>0$ from *c* such that a geodesic ray through this point, at right angles to $\beta _3$ in the half-space that $\beta _5$ points into, is parallel to $\beta _5$. This point can be found because the geodesic through it forms a triangle, together with segments of $\beta _3$ and $\beta _5$, that has one ideal vertex and angles of $\pi /2$ and $\gamma$ (as defined in equation (5) above) at its other two angles. The distance *z* satisfies $\cosh z = 1/\sin \gamma$, see (Ratcliffe (2019), Thrm. 3.5.7). Again fixing $\ell$, and regarding $\gamma$ and hence $z = z(\gamma )$ as functions of *x*, we find that since $\gamma$ is a decreasing function of *x*, then *z* is an increasing function of *x*.

As $x\rightarrow 0^+$, $\gamma \rightarrow \pi /2$, $y\rightarrow \ell$, and $z\rightarrow 0$; as $x\rightarrow X_1$, $y\rightarrow 2\ell$. Thus $y+z$ is less than $2\ell$ for *x* near 0 and greater than $2\ell$ for *x* near $X_1$. Since *y* and *z* each increase with *x* their sum does as well, so there is a unique $x=X_0$ with $0<X_0<X_1$ for which $y+z = 2\ell$, and for all $x\in (X_0,X_1)$, $y< 2\ell < y+z$. By the equations above and the angle addition law for hyperbolic cosine, $X_0$ satisfies:

$$\begin{aligned} \cosh (2\ell ) = \frac{\cosh \ell +\sinh \ell \sinh X_0\cosh X_0}{1-\sinh ^2 X_0\sinh ^2\ell } \end{aligned}$$

Now given $x\in (X_0,X_1)$, let $\alpha _1$, $\alpha _2$, and $\alpha _3$ be geodesic arcs as described above, with lengths $\ell$, *x*, and $2\ell$, respectively; let $v_1 = \alpha _1\cap \alpha _2$ and $v_2 = \alpha _2\cap \alpha _3$; and let $\beta _5$ be the geodesic ray described above with its endpoint at the other endpoint $v_5$ of $\alpha _1$. Because $2\ell < y+z$, a geodesic ray from the other endpoint $v_3$ of $\alpha _3$, at right angles to $\alpha _3$ and pointing into the same half-space bounded by the geodesic containing it as $\beta _5$, does intersect $\beta _5$. Let $\alpha _4$ be the arc contained in this ray and joining $v_3$ to its point $v_4$ of intersection with $\beta _5$. Finally, let $\alpha _5$ be the arc of $\beta _5$ joining $v_4$ back to $v_5$. This completes the construction of the self-intersecting pentagon. $\square$

### Proposition 3.2

For any data $(o,\textbf{v},s,\theta ,\ell )$ of a walk with jumps model such that the jump angle $\theta = \pi /2$, there exist $0<X_0<X_1$ (as defined above) such that for any $x\in (X_0,X_1)$ and $T_0>0$, there exist two walks with jumps in the model such that:

- the first has duration $T\ge T_0$ and a single jump at time 0; and
- the second has duration $T_1 < T$, its first jump (a burst of two) at time *x*/*s*, and a total number of jumps that increases linearly with $T_0$.

More precisely: there exists $K>0$, depending only on *x*, such that $T\in [T_0,T_0+K)$, and $b = b(x) >0$ such that the second walk’s jump times $(t_1,t_2,\hdots ,t_n)$ satisfy $t_1 = x/s$, $t_2<K$, and $t_i = t_2+(i-2)b$ for each $i\ge 2$.

### Proof

For the value of $\ell$ in the given initial data, let $0<X_0 < X_1$ be as in Lemma 3.1. Then for $x\in (X_0,X_1)$, arrange the pentagon given by that result so that its vertex $v_1$ is at *o* and $\textbf{v}$ points along the side $\alpha _2$ of length *x* toward $v_2$. Then the side $\alpha _1$ of length $\ell$ is counterclockwise from $\alpha _2$ at $v_1 = o$. As in the proof of Lemma 3.1, let $\beta _5$ be the ray sharing the other endpoint $v_0$ of $\alpha _1$ and containing the pentagon’s side $\alpha _5$. Since the first walk with jumps has a single jump at time 0, which has length $\ell$, its sole nontrivial walk segment is contained in $\beta _5$.

**Fig. 5:**
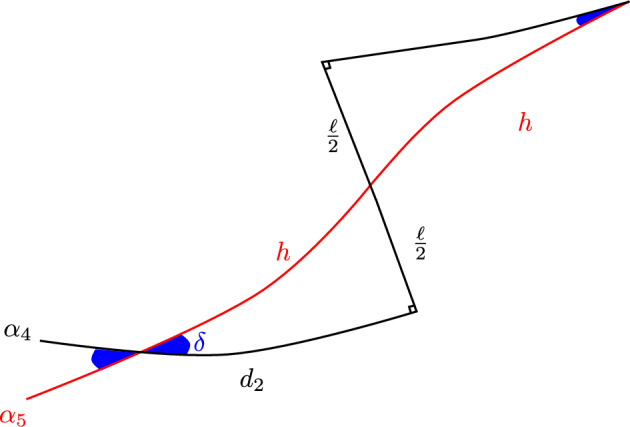
The triangle at the tip of the pentagon

Since the second walk is prescribed to have a burst of two jumps at $t_1 = x/s$, its first walk segment is $\alpha _2$, its first jump segment is $\alpha _3$, and its second walk segment is contained in the geodesic ray $\beta _4$ that has an endpoint at $v_3$ and contains $\alpha _4$. We prescribe the second jump time $t_2$ to be larger than $\ell _4/s$, where $\ell _4$ is the length of $\alpha _4$—so the second walk segment contains $\alpha _4$—and with the property that the second jump axis is bisected by its intersection with $\beta _5$. Precisely: taking $d_2 = st_2 - \ell _4$, we must have

6 $$\begin{aligned} \cosh d_2 = \frac{\sqrt{\sinh ^2(\ell /2)+\sin ^2\delta }}{\cosh (\ell /2)\sin \delta }, \end{aligned}$$

where $\delta$ is the pentagon’s angle at its vertex $v_4$. This follows from the hyperbolic laws of sines and cosines; see Figure 5. There, the hyperbolic law of cosines gives that $\cosh h = \cosh d_2 \cosh (\ell /2)$, and the hyperbolic law of sines that $\sinh h = \sinh (\ell /2)/\sin \delta$. Substituting for *h* in the first equation yields the formula (6).

If we now prescribe for each $i>2$ that $t_i - t_{i-1} = b \doteq 2d_2/s$, then the *i*th jump segment is also bisected by its intersection with $\beta _5$ for each such *i*. To see this, rotate the triangle of Figure 5 by 180 degrees about its vertex of intersection between the sides of length *h* and $\ell /2$: the resulting isometric copy also has its side of length *h* in $\beta _5$, its side of length $\ell /2$ is the other half of the second walk’s second jump segment, and its side of length $d_2$ is the first half of the third walk segment. Rotating this triangle $180^{\circ }$ around its other non right-angled vertex yields the second half of the third walk segment and the first half of the third jump segment, and we proceed further by rotating around subsequent non right-angled vertices.

We now take $$K = (\ell _5 + 2h)/s$$, where $$\ell _5$$ is the length of the pentagon’s side $$\alpha _5$$ and *h* is as in Figure 5. Then for any $$T_0>0$$, there is a walk with given initial data, a single jump at time 0, and duration in $$[T_0, T_0+K)$$ that terminates at one of the intersection points of $$\beta _5$$ with a walk segment described in the paragraph above. These are segments of the second walk with jumps, which we run for as long as it takes to meet the first. $$\square $$

Note that the above construction of a specific pair of walks that have a common endpoint, has a biological interpretation: one way in which the endpoint can fail to faithfully encode the jump times is that a jump at time zero can be mimicked by a sequence of jumps at later times.

We now record an observation that will allow us to create “rotationally symmetric” walks with jumps.

### Lemma 3.3

For a walk with jumps in a model with data $(o,\textbf{v},s,\theta ,\ell )$, having duration *T*, if the jump times $(t_1,\hdots ,t_{2n})$ and burst vector $(j_1,\hdots ,j_{2n})$ are symmetric in the sense that $T/2 - t_i = t_{2n+1-i} - T/2$ and $j_i = j_{2n+1-i}$ for each $i\le n$, then:

- the associated walk with jumps path is itself symmetric under $\pi$-rotation around the walk’s midpoint;
- denoting this rotation as $\rho$, the walk’s endpoint and endvector are respectively $\rho (o)$ and $d\rho _o(-\textbf{v})$; and
- the word *w* supplied by Lemma 2.7 that carries $(o,\textbf{v})$ to the endpoint-endvector pair $(p,\textbf{w})$ of the walk with jumps satisfies $\rho w\rho = w^{-1}$.

### Proof

We call the *i*th walk segment (as in Definition 1.5) $\alpha _i$, for $1\le i\le 2n+1$, and the *j*th jump segment $\beta _j$ for $1\le j\le 2n$. Note that $\alpha _i$ has length $\ell _i = s \delta _i$ for $1< i \le 2n$, where $\delta _i = t_i - t_{i-1}$; and $\ell _1 = s t_1$ and $\ell _{2n+1} = s(T - t_{2n})$. By hypothesis:

$$\begin{aligned} T/2-t_1 = t_{2n} - T/2,\quad \Rightarrow \quad T - t_{2n} = t_1,\quad \Rightarrow \quad \ell _1 = \ell _{2n+1}. \end{aligned}$$

Similarly, for $1<i\le n$, $\ell _i = \ell _{2n+2-i}$ since:

7 $$\begin{aligned} \delta _i&= \left[ (T/2 - t_i) - (T/2 - t_{i-1})\right] \\&= \left[ (t_{2n+1-i} - T/2) - (t_{2n+1 - (i-1)} - T/2)\right] = \left[ t_{2n+2-i} - t_{2n+1-i}\right] = \delta _{2n+2-i}.\nonumber \end{aligned}$$

The symmetry hypothesis on the jump vector similarly implies that $\beta _j$ and $\beta _{2n+1-j}$ have identical lengths for each *j*.

It follows from this that the midpoint of the associated walk with jumps path is the midpoint of $\alpha _{n+1}$, so the rotation $\rho$ by an angle $\pi$ around this point takes $\alpha _{n+1}$ to itself. By construction of the walk with jumps, each of $\beta _n$ and $\beta _{n+1}$ intersects $\alpha _{n+1}$ at an angle of $\pi -\theta$ clockwise from the tangent vector pointing into $\alpha _{n+1}$ at their point of intersection. Since $\rho$ exchanges the endpoints of $\alpha _{n+1}$, and $\beta _n$ and $\beta _{n+1}$ have identical lengths, it also exchanges $\beta _n$ and $\beta _{n+1}$. Now $\alpha _n$ and $\alpha _{n+2}$ respectively intersect $\beta _n$ and $\beta _{n+1}$, each at an angle of $\pi -\theta$ *counter*clockwise from the tangent vector pointing into $\beta _n$ or $\beta _{n+1}$, so $\rho$ exchanges $\alpha _n$ with $\alpha _{n+2}$ since $\ell _n = \ell _{n+2}$.

Working our way outward from the midpoint as above, we ultimately find that $\rho$ takes the entire walks with jumps path to itself. The assertion about the walk’s endpoint and endvector now follow from this and Remark 1.6.

We now interpret the symmetry condition in terms of the word *w* supplied by Lemma 2.7 that carries $(o,\textbf{v})$ to the endpoint-endvector pair $(p,\textbf{w})$ of the walk with jumps. For this walk, *w* has the form

$$\begin{aligned} w = g_{t_1}f^{j_1}g_{\delta _2}f^{j_2}\hdots g_{\delta _{2n}}f^{j_{2n}}g_{T-t_{2n}}, \end{aligned}$$

where for each $i>1$, $\delta _i = t_i - t_{i-1}$ is the difference between successive jump times defined above. From (7) we have for each such *i* that $\delta _i = \delta _{2n+2-i}$. In particular, $\delta _{n+2} = \delta _n$, $\delta _{n+3} = \delta _{n-1}$, and so forth through $\delta _{2n} = \delta _2$. Also using that $T-t_{2n}=t_1$, we obtain the following visibly symmetric form for *w*:

$$ w = \left( g_{t_1}f^{j_1}g_{\delta _2}\hdots g_{\delta _n}f^{j_n}\right) g_{\delta _{n+1}} \left( f^{j_n}g_{\delta _n}\hdots g_{\delta _2}f^{j_1}g_{t_1}\right) $$

We now write $w = w_0 w_1$ where $w_0 = g_{t_1}f^{j_1}g_{s_2}\hdots g_{s_n}f^{j_n}g_{s_{n+1}/2}$ is its “first half” and $w_1 = g_{s_{n+1}/2}f^{j_n}g_{s_n}\hdots g_{s_2}f^{j_1}g_{t_1}$ its second. By Lemma 2.7, $w_0$ carries the origin *o* to the walk’s midpoint. Therefore the rotation $\rho$ by $\pi$ about this point has the form $w_0 \rho _0 w_0^{-1}$, where $\rho _0$ is the $\pi$-rotation about *o*. Recalling that a $\pi$-rotation is its own inverse, we consider the conjugate of *w* by $\rho$:

$$\begin{aligned} \rho w \rho ^{-1}&= \left( w_0\rho _0w_0^{-1}\right) w_0w_1 \left( w_0\rho _0 w_0^{-1}\right) \\&= w_0 \rho _0 w_1 w_0 \rho _0 w_0^{-1} = w_0 (\rho _0 w_1 \rho _0)(\rho _0 w_0\rho _0) w_0^{-1} \end{aligned}$$

We claim now that $\rho _0 w_i \rho _0 = w_{1-i}^{-1}$ for $i = 0$ and 1, from which it will follow that $\rho w\rho = w^{-1}$ using the above. This uses the fact that $\rho _0$ conjugates *f* to $f^{-1}$, and likewise $g_t$ for any $t\in \mathbb {R}$, since their axes run through its fixed point *o*. (One can check this fact directly by taking $o = \textbf{0}$ and $\textbf{v}= \textbf{e}_1$ in the Poincaré disk model and using the explicit descriptions below.)

$$ \rho _0 = \left( {\begin{smallmatrix} i & 0 \\ 0 & -i \end{smallmatrix}}\right) \quad \hbox {and}\quad g_t = \left( {\begin{smallmatrix} \cosh (t/2) & \sinh (t/2) \\ \sinh (t/2) & \cosh (t/2) \end{smallmatrix}}\right) $$

Now inserting $\rho _0\rho _0$, a form of the identity, between each syllable of $w_0$ for instance, we obtain:

$$\begin{aligned} \rho _0w_0\rho _0&= \left( \rho _0g_{t_1}\rho _0\right) \left( \rho _0f^{j_1}\rho _0\right) \left( \rho _0g_{s_2}\rho _0\right) \hdots \left( \rho _0g_{s_{n+1}/2}\rho _0\right) \\&= g_{t_1}^{-1} f^{-j_1} g_{s_2}^{-1}\hdots g_{s_{n+1}/2}^{-1} = w_1^{-1} \end{aligned}$$

Likewise for $w_1$, and the claim, and hence the lemma, holds. $\square$

We now exploit Lemma 3.3 to promote Proposition 3.2’s walks with jumps to a pair sharing an endpoint *and endvector*.

### Proposition 3.4

For any data $(o,\textbf{v},s,\theta ,\ell )$ of a walk with jumps model such that the jump angle $\theta = \pi /2$, there exist $b,K>0$ and $0<X_0<X_1$ such that for any $T_0>0$ and $x\in (X_0,X_1)$, there exist two walks with jumps in the model that have a common endpoint and endvector; and such that:

- The first has duration $T_2\in [2T_0,2T_0+2K)$ and two jumps, at time 0 and $t=T_2$, with burst vector (1, 1);
- The second has jump times $(t_1,\hdots ,t_{2n})$ and burst vector $(2,1,\hdots ,1,2)$ for some $n\ge 2$, where $t_1 = x/s$, $t_{2n} = 2T_1 - t_1$ and for $2\le i\le n$, $t_i = t_2+(i-2)b$ and $t_{2n+1-i} = 2T_1 - t_i$, where $2T_1$ is its duration.

### Proof

For the given initial data, let $0< X_0 < X_1$ be supplied by Proposition 3.2. Then for $T_0>0$ and $x\in (X_0,X_1)$ let that result supply a duration *T* for a first walk and jump times $(t_1,t_2,\hdots t_n)$ for a second, with burst vector $(2,1,\hdots ,1)$ such that the two walks with jumps share an endpoint. Let $T_1$ be the duration of the second walk with jumps supplied by Proposition 3.2.

We construct the walks with jumps for the current result to be rotationally symmetric in the sense of Lemma 3.3, with those supplied by Proposition 3.2 as initial segments (up to their midpoints). Thus we take the first to have duration $T_2 \doteq 2 T$ and single jumps at times 0 and $T_2 = T_2-0$; and the second to have duration $2T_1$ and jump times and burst vector as given in the statement. This walk’s jump data is thus symmetric, i.e.,  the jump times satisfy the equation $T_1 - t_i = t_{2n+1-i} - T_1$ for each *i*, and the burst vector entries also match, so by Lemma 3.3 its associated walk-with-jumps path is symmetric by the rotation $\rho$ about its midpoint.

The first walk with jumps also has symmetric jump data, so its walk-with-jumps path is also rotationally symmetric. But these two paths have the same midpoint, since by construction their initial segments are the walks with jumps given by Proposition 3.2 and these have a common endpoint. Hence the current walks with jumps have both a common endpoint $\rho (o)$ and endvector $d\rho _o(-\textbf{v})$. $\square$

The walks constructed in Proposition 3.4 have different durations and numbers of jumps, but this can be fixed by reversing their roles and doubling again.

### Proposition 3.5

For any data $(o,\textbf{v},s,\theta ,\ell )$ of a walk with jumps model such that the jump angle $\theta = \pi /2$, there exist $b,K>0$ and $0<X_0<X_1$ such that for any $T_0>0$ and $x\in (X_0,X_1)$, there exist two walks with jumps in the model that have identical durations $T_2 + 2T_1$—for $T_2\in [2T_0,2T_0+2K)$ and some $T_1>0$—numbers of jumps, and endpoint and endvector; and such that:

- The first has jump times $(0,T_2,T_2+t_1,\hdots ,T_2+t_{2n})$, for some $n\ge 2$, and burst vector $(1,1,2,1,\hdots ,1,2)$;
- The second has jump times $(t_1,\hdots ,t_{2n},2T_1,2T_1+T_2)$, for the same *n*, and burst vector $(2,1,\hdots ,1,2,1,1)$.

In each case above, $t_1 = x/s$, $t_{2n} = 2T_1 - t_1$ and for $2\le i\le n$, $t_i = t_2+(i-2)b$ and $t_{2n+1-i} = 2T_1 - t_i$.

### Proof

To construct the first and second walks here we use the corresponding walks from Proposition 3.4 as their initial segments, so that result supplies $T_2$, $T_1$, and the jump times $t_1,\hdots ,t_n$. The initial segments thus have the same endpoint and endvector, after a duration of $T_2$ for the first and $2T_1$ for the second. For the terminal segment of the first walk here, we then use a copy of the *second* walk supplied by Proposition 3.4; and conversely, for the terminal segment of the second walk we use a copy of the previous Proposition’s *first* walk. $\square$

### Remark 3.6

The quantity $X_1 \doteq \cosh ^{-1}(\coth \ell \tanh (2\ell ))$ above, which is from Lemma 3.1, decreases to 0 as $\ell \rightarrow \infty$. Thus the lag between the two walks’ first jump times allowed by the hypotheses of the results in this section decreases to 0 as either of the model parameters $\ell$ and *s* increase without bound.

## Consequences of negative curvature leading to distinct endpoints

In this section, a counterpoint to the previous one, we show that both the pattern and number of jumps can affect the endpoint of a walk with jumps. First, in Section 4.1, we give a criterion for producing walks with identical initial data, duration, and number of jumps but different endpoints. We subsequently prove quasigeodesicity of walk-with-jumps paths, in Sect. 4.2, a result that has as a particular consequence that having a different enough number of jumps leads to different endpoints.

### Future quadrants and points of no return

#### Lemma 4.1

Let $(o,\,\textbf{v},\,s,\,\theta ,\,\ell )$ be the data of a walk with jumps model. For a walk with jumps in the model, its walk axes $\gamma _i$ from Definition 2.6 are pairwise disjoint. Furthermore if $j<i<k$, then $\gamma _j$ and $\gamma _k$ lie in distinct components of $\mathbb {H}^2\setminus \gamma _i$. For each *i*, the shortest distance *d* from $\gamma _i$ to $\gamma _{i+1}$ satisfies $\sinh (d/2) = \sin \theta \sinh (\ell /2)$.

The jump axes $\lambda _i$ are also pairwise disjoint and satisfy the analogous separation property; and for $i>1$, the shortest distance $x_i$ from $\lambda _{i-1}$ to $\lambda _i$ satisfies $\sinh (x_i/2) = \sin \theta \sinh (s(t_i-t_{i-1})/2)$.

**Fig. 6:**
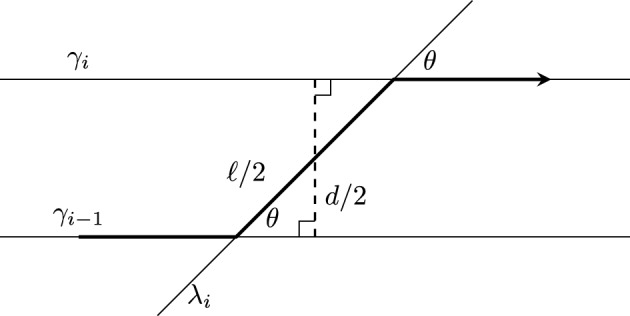
Two walk axes $\gamma _{i-1}$ and $\gamma _i$, and a jump axis $\lambda _i$, with a segment of a walk-with-jumps path in bold. The shortest distance between walk axes is along the dashed arc

#### Proof

We can take the $\gamma _i$ and $\lambda _j$ to be oriented “in the direction of travel” (i.e., in the direction that the relevant conjugate of the $g_t$ or *f* translates). For a fixed $i>0$, $\gamma _{i-1}$ and $\gamma _i$ both intersect $\lambda _i$. At each intersection, the angle from its positive direction to that of $\lambda$ is equal to $\theta$. If $\gamma _i$ and $\gamma _{i-1}$ had a point of intersection they would thus form a triangle, together with $\lambda _i$, whose angles at the edge contained in $\lambda _i$ were $\theta$ and $\pi -\theta$. This would contradict that the angle sum of a hyperbolic triangle with at least one compact vertex is less than $\pi$.

The same argument also rules out $\gamma _i$ and $\gamma _{i-1}$ sharing an ideal endpoint, so they are at a nonzero distance from each other. The minimum distance *d* is attained at endpoints of a unique geodesic arc that meets each of $\gamma _{i-1}$ and $\gamma _i$ at right angles.

To determine *d*, we first note that the $\pi$-rotation around the midpoint of the arc of $\lambda _i$ that joins $\gamma _{i-1}$ to $\gamma _i$ takes $\lambda _i$ to itself and exchanges $\gamma _{i-1}$ with $\gamma _i$. (This can be seen from the fact that it interchanges their tangent vectors at their points of intersection with $\lambda _i$, which determine the geodesics.) The geodesic arc joining $\gamma _{i-1}$ to $\gamma _i$ is therefore also preserved by this rotation, so it contains its fixed point as a bisector.

The formula for *d* now follows from the hyperbolic law of sines, see Figure 6. The distance $x_i$ between $\lambda _{i-1}$ and $\lambda _i$ is established analogously, since these geodesics are joined by an arc of $\gamma _i$ of length $s(t_i-t_{i-1})$. (The main difference from the previous case being that this depends on *i*, since the time intervals between jumps can vary)

We finally note that by construction of the walk with jumps, the segment of $\lambda _{i}$ joining $\gamma _{i-1}$ to $\gamma _i$ is on the opposite side of $\gamma _i$ from the arc of $\lambda _{i+1}$ joining $\gamma _i$ to $\gamma _{i+1}$ (for $0<i<n$). From this, it follows that $\gamma _i$ separates $\gamma _{i-1}$ from $\gamma _{i+1}$. A quick inductive argument now ensures for all $j<i<k$ that $\gamma _j$ and $\gamma _k$ lie in opposite complementary components of $\gamma _i$. $\square$

#### Corollary 4.2

Let $(o,\,\textbf{v},\,s,\,\theta ,\,\ell )$ be the data of a walk with jumps model. For a walk with jumps in the model, the parametrization of the associated walk-with-jumps path given in Definition 1.5 defines an embedding of the parameter interval to $\mathbb {H}^2$.

For each $i\ge 1$, any points *a*, $p_i$, and *b* on the walk-with-jumps path such that the parameter value of *a* (respectively, *b*) is less (resp. greater) than that of $p_i$ determine a triangle in $\mathbb {H}^2$ whose interior angle at $p_i$ is at least $\pi -\theta$. The same angle bound likewise holds for an analogous triangle with vertices at *a*, $q_i$, and *b*.

#### Proof

Since the parametrization given in Definition 1.5 is by arclength on any sub-interval of the parameter interval that maps to a walk or jump segment, it is injective on such sub-intervals. Moreover, walk and jump segments that share an endpoint do not intersect outside this endpoint, since it is a general property of hyperbolic geodesics that they intersect in at most a single point. Therefore for parameter values *t* and $t'\ne t$ mapping to the same point, we may assume that they do not belong to the same or adjacent sub-intervals as above. However then the walk or jump segments that they map to, which are not identical and do not share an endpoint, are separated by the walk or jump axis containing any segment that lies between these two on the walk-with-jumps path, by Lemma 4.1.

**Fig. 7:**
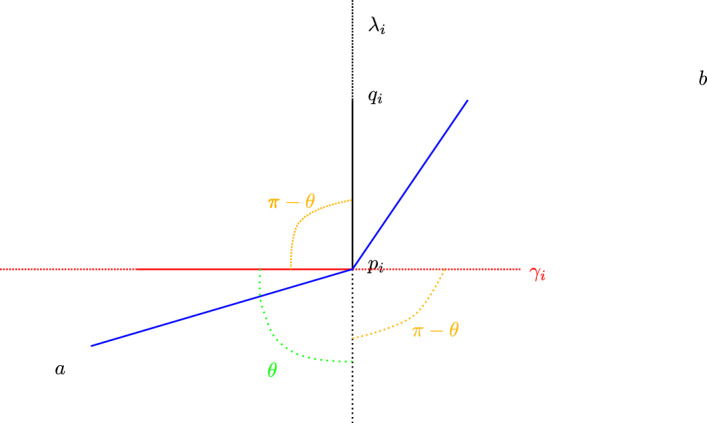
To accompany Corollary 4.2

We address the triangle $\triangle$ with vertices at *a*, $p_i$, and *b*; the other case is similar. By Lemma 2.7, *o* lies on $\gamma _1$, $p_i$ on $\gamma _i\cap \lambda _i$, and $q_i$ on $\lambda _i\cap \gamma _{i+1}$. Thus by Lemma 4.1, the edge of $\triangle$ joining *a* to $p_i$ lies entirely in the half-plane bounded by $\gamma _{i}$ that does not contain $q_i$ and the half-plane bounded by $\lambda _i$ that does not contain $p_{i+1}$. These half-planes have angle of intersection $\theta$ at $p_i$, so the interior angle of *T* here exceeds the complementary angle $\pi -\theta$, see Figure 7.$\square$

We begin by formally defining a notion that has appeared already in the proof of Lemma 2.7.

#### Definition 4.3

Let $(o,\,\textbf{v},\,s,\,\theta ,\,\ell )$ be the data of a walk with jumps model, and suppose a walk with jumps in the model is specified by a duration *T*; jump time sequence $(t_1,\hdots ,t_n)$; and burst vector $(j_1,\hdots ,j_n)$. For any $T_0\le T$, the *duration-*$T_0$ *initial segment* of the given walk with jumps is the one sharing the same initial data; with duration $T_0$; sequence of jump times $(t_1,\hdots ,t_k)$, where $k\le n$ is the largest index such that $t_k\le T_0$; and burst vector $(j_1,\hdots ,j_k)$ for the same *k*. Conversely, we say that the original walk with jumps is obtained by *prolonging* any initial segment.

#### Remark 4.4

Let $f,g_t\in \operatorname {PSL}_2(\mathbb {R})$ be associated to a walk with jumps model as in Lemma 2.1, and let $w\in \operatorname {PSL}_2(\mathbb {R})$ be the word in syllable form associated to a walk with jumps in the model by Definition 2.6. For any initial segment of this walk with jumps, the syllable form of its associated word $w_0\in \operatorname {PSL}_2(\mathbb {R})$ is a prefix of *w*. Specifically, $w=w_0w_1$ for some $w_1$, where the syllable form of *w* can be determined from the syllable forms of $w_0$ and $w_1$ by replacing the last syllable $g_{s_0}$ of $w_0$ and the first syllable $g_{s_1}$ of $w_1$ with a single syllable $g_{s_0+s_1}$.

The result below is another consequence of Lemma 4.1. Given a bi-infinite geodesic $\gamma \subset \mathbb {H}^2$ such as one of the walk or jump axes of a walk with jumps, a *half-plane bounded by* $\gamma$ is the closure of one component of $\mathbb {H}^2-\gamma$. Its *complementary* half-plane is the closure of the other component.

#### Corollary 4.5

Let $(o,\,\textbf{v},\,s,\,\theta ,\,\ell )$ be the data of a walk with jumps model, and suppose a walk with jumps in the model is specified by a duration *T*; jump time sequence $(t_1,\hdots ,t_n)$, $n\ge 1$; and burst vector $(j_1,\hdots ,j_n)$. Let $\gamma _g$ and $\lambda _f$ be as in Lemma 2.1, and let $\gamma _i$, for $i\in \{1,\hdots ,n+1\}$, and $\lambda _j$, for $j\in \{1,\hdots ,n\}$, be the walk and jump axes from Definition 2.6. Then:

- Let $H_0^+$ be the half-plane bounded by $\gamma _g = \gamma _1$ that contains $\gamma _2$ and $K_0^+$ the half-plane bounded by $\lambda _f$ that contains $\lambda _1$. Then $H_0^+\cap K_0^+$ contains the entire associated walks-with-jumps path.
- For each $i\in \{1,\hdots ,n\}$ let $H_i^-$ be the half-plane bounded by $\gamma _{i+1}$ that contains *o* and $K_i^-$ the half-plane bounded by $\lambda _i$ that contains *o*. The entire walks-with-jumps path of the duration-$t_i$ initial segment is contained in $H_i^-\cap K_i^-$, and the remainder of the full walk-with-jumps path is contained in the intersection of the complementary half-planes $H_i^+\cap K_i^+$.

Moreover, taking $\lambda _{n+1}$ to be the geodesic through the endpoint of the walk with jumps at an angle of $\theta$ to $\gamma _{n+1}$, measured counterclockwise from the endvector, there is a half-plane $K_{n+1}^-$ bounded by $\lambda _{n+1}$ such that $H_n^-\cap K_{n+1}^-$ contains the entire walk-with-jumps path.

#### Proof

We claim that $H_0^+$ contains each axis $\gamma _i$ for $i\ge 1$. Of course $H_0^+$ contains its boundary $\gamma _1$, and the case $i=2$ holds by definition. Now for $i\ge 2$, supposing that $\gamma _j\subset H_0^+$ for each $j\le i$, we claim that $\gamma _{i+1}$ is also contained in $H_0^+$. This follows from Lemma 4.1, which asserts that $\gamma _i$ separates $\gamma _{i+1}$ from $\gamma _{i-1}$, so since $\gamma _{i-1}$ and $\gamma _i$ lie in $H_0^+$, so does $\gamma _{i+1}$. The claim thus follows by induction.

By Lemma 2.7, it now follows that $H_0^+$ contains each walk segment of the associated walk-with-jumps-path. And since each jump segment is a geodesic arc joining endpoints of two walk segments, and $H_0^+$ is convex, it also contains each jump segment. Therefore $H_0^+$ contains the entire associated walk-with-jumps path. An entirely analogous argument shows that $K_0^+$ does as well, and the first bulleted claim is proved.

We now prove the second claim. We claim that if $j\le i+1$, then $\gamma _j$ is in $H_i^-$, the half plane bounded by $\gamma _{i+1}$ that contains *o*. Indeed, if $j=i+1$ the result is clear, so we assume $j<i+1$. In the special case, $j=0$, Lemma 4.1 implies $\gamma _0$ is contained in a distinct half plane bounded by $\gamma _0$ and $\gamma _0$ contains *o*. For $0<j<i+1$, Lemma 4.1 implies that $\gamma _j$ separates $o\in \gamma _0$ from $\gamma _j$. Therefore, the half plane bounded by $\gamma _{i+1}$ that contains $\gamma _j$ must be the one that contains *o*.

If *x* lies in the duration–$t_i$ in initial segment, then *x* either lies on one of the $\gamma _j$ for $j<i+1$ or lies on a geodesic between $\gamma _{j_1},\gamma _{j_2}$ where $j_1,j_2\le i+1$. In the first case, we immediately have that *x* lies in $H_i^-$, and in the second *x* lies in $H_i^-$ by convexity of half planes. A similar argument shows that *x* also lies in $K_i^-$. Then $x\in H_i^-\cap K_i^-$.

On the other hand, if *y* lies in the remainder of the full walk-with-jumps path, then *y* lies on some $\gamma _j$ for $j\ge i+1$ or *y* lies on a geodesic between $\gamma _{j_1},\gamma _{j_2}$ where $j_1,j_2\ge i+1$. Lemma 4.1 implies that if $j> i+1$, then $\gamma _{i+1}$ separates $\gamma _j$ from $\gamma _0$ which contains *o*. Therefore, for $j\ge i+1$, $\gamma _j$ lies in $H_i^+$, the half plane bounded by $\gamma _{i+1}$ that does not contain *o*. Therefore, $y\in H_i^+$ by convexity of $H_i^+$. A similar argument shows *y* also lies in $K_i^+$. Therefore, $y\in H_i^+\cap K_i^+$.

We now address the Corollary’s final claim. Note that each of $\lambda _{n+1}$ and $\lambda _n$ intersect $\gamma _{n+1}$ at an angle of $\theta$: the former by construction, and the latter as observed in Lemma 4.1. They coincide if $t_n = T$; otherwise they do not intersect (also as observed in Lemma 4.1). In the former case, take $K_{n+1}^- = K_n^-$. In the latter, let $K_{n+1}^-$ be the half-plane bounded by $\lambda _{n+1}$ and containing $\lambda _n$. Then $K_{n+1}^-$ contains the $(n+1)$st walk segment, since the endpoints of this segment lie on $\lambda _{n+1}$ and $\lambda _n$, and it contains the half-plane $K_n^-$ opposite this segment. Therefore by the previous claim, $K_{n+1}^-$ contains the entire walk-with-jumps path. $\square$

#### Definition 4.6

Motivated by the second bullet of Corollary 4.5, for $H_n^+$, $\lambda _{n+1}$, and $K_{n+1}^-$ as defined there, we call $K_{n+1}^+$ the half-plane bounded by $\lambda _{n+1}$ opposite $K_{n+1}^-$ and deem the intersection $H_n^+\cap K_{n+1}^+$ of half-planes the *future quadrant* of the walk with jumps specified there.

### Quasigeodesicity

In this section we will show that the walk-with-jumps paths in $\mathbb {H}^2$ from Definition 1.5 are “quasi-geodesic”. This property is well known to have important consequences in the study of negatively curved spaces. A key consequence for our application, recorded in Corollary 4.13 below, is that walks with different (enough) *numbers* of jumps have different endpoints, irrespective of any other consideration. As in the previous subsection, our results here are *effective*, meaning that we produce explicit constants.

#### Definition 4.7

For a fixed $\lambda \in (0,1]$ and $\epsilon \ge 0$, a $(\lambda ,\epsilon )$*-quasi-geodesic* in a metric space (*X*, *d*) is a map $c: I\rightarrow X$, where $I\subset \mathbb {R}$ is an interval, such that for all $t,t'\in I$:

$$\begin{aligned} \lambda |t-t'| - \epsilon \le d(c(t),c(t')) \le \frac{1}{\lambda } |t-t'| + \epsilon . \end{aligned}$$

This definition asserts that the path *c* does not shrink or expand distance by more than a multiplicative factor of $\lambda$ and an additive factor of $\epsilon$. In applying the notion to a walk-with-jumps path we will leverage the fact that it is a broken geodesic. Our parametrization described in Definition 1.5 is by arclength on each piece, so the right-hand inequality above will automatically hold for any $\lambda \in (0,1]$ and $\epsilon \ge 0$.

#### Proposition 4.8

(Hyperbolic Law of Cosines, (Bridson and Haefliger (1999), I.2.7)). Let $\triangle$ be a hyperbolic triangle with vertices *A*, *B*, *C*. Let $a = d(B,C)$, $b=d(C,A),$ and $c=d(A,B)$. Let $\theta$ denote the vertex angle at *C*. Then

$$\begin{aligned} \cosh c =\cosh a\cosh b - \sinh a\sinh b \cos \theta \end{aligned}$$

In particular, if $|\theta |= \frac{\pi }{2}$, then:

$$\begin{aligned} \cosh c = \cosh a\cosh b \end{aligned}$$

#### Lemma 4.9

For a triangle $\triangle \subset \mathbb {H}^2$ with vertices *A*, *B*, and *C* and opposite side lengths *a*, *b*, and *c* respectively, if $\triangle$ has interior angle $\theta$ at *C* then

$$\begin{aligned} a + b - c < \delta _{\theta } \doteq \ln \left( \frac{2}{1-\cos \theta }\right) . \end{aligned}$$

This bound is sharp, not attained, but asymptotically approached as $a = b\rightarrow \infty$.

#### Remark 4.10

We fix $\theta = \pi /2$ to illustrate the strong contrast between $\mathbb {R}^2$ and $\mathbb {H}^2$. By the result above, for a right triangle in $\mathbb {H}^2$ with hypotenuse of length *c*, the difference $a+b-c$ is universally bounded above by $\ln 2$. On the other hand, for an isosceles right triangle in $\mathbb {R}^2$ with shorter side length $a=b$ the difference $a+b-c = (2-\sqrt{2})a$ increases linearly with *a*.

#### Proof

Using the hyperbolic law of cosines from Proposition 4.8, we consider $a+b - c$ as a function $f_{\theta }(a,b)$ on the quadrant $\{a,b\ge 0\}$ in the *ab*-plane by substituting

$$\begin{aligned} c = \cosh ^{-1}(\cosh a\cosh b - \sinh a\sinh b \cos \theta ). \end{aligned}$$

A somewhat messy calculus computation gives that the gradient vector $\nabla f_{\theta }(a,b)$ is a positive scalar multiple of $(\sinh ^2 B,\sinh ^2 A)$. Thus at any $(a,b) \ne (0,0)$ (the global minimum of $f_{\theta }$), the gradient points away from the origin and toward the diagonal (*x*, *x*). So for instance, for any fixed $X>0$, the maximum of $f_{\theta }$ on $[0,X]^2$ occurs at (*X*, *X*). Moreover, $f_{\theta }(x,x)$ is an increasing function of *x*, and it follows that the values of $f_{\theta }$ on the entire quadrant are bounded above by $\delta _{\theta } = \lim _{x\rightarrow \infty } f_{\theta }(x,x)$.

To evaluate the limit, we exponentiate $f_{\theta }(x,x)$ and use the fact that $\cosh ^{-1}y = \ln \left( y+\sqrt{y^2-1}\right)$ to write

$$\begin{aligned} e^{f_{\theta }(x,x)} = \frac{e^{2x}}{\cosh ^2x - \sinh ^2x\cos \theta + \sqrt{(\cosh ^2x - \sinh ^2x\cos \theta )^2 - 1}} \end{aligned}$$

Multiplying top and bottom by $e^{-2x}$ then evaluating the limit and taking a natural log yields the formula for $\delta _{\theta }$ given above. $\square$

#### Proposition 4.11

Let $(o,\,\textbf{v},\,s,\,\theta ,\,\ell )$ be the data of a walk with jumps model. For a walk with jumps in the model specified by duration *T*, jump times $(t_1,\hdots ,t_n)$, and burst vector $(j_1,\hdots ,j_n)$, the distance *d* from *o* to the walk’s endpoint satisfies

$$\begin{aligned} sT + \ell N \ge d \ge sT + \ell N - 2n\delta _{\pi -\theta }, \end{aligned}$$

for $\delta _{\theta }$ as in Lemma 4.9, where $N = \sum _{i=1}^n j_i$ is the number of jumps. Moreover, supposing that $\delta _{\pi -\theta } < \ell /2$, define

8 $$\begin{aligned} \lambda = \frac{\ell - 2\delta _{\pi -\theta }}{\ell }. \end{aligned}$$

Then the associated walk-with-jumps path is a $(\lambda ,2\delta _{\pi -\theta })$-quasi-geodesic.

#### Remark 4.12

The length lower bound above implies that “more bursty” walks are closer to geodesic. To support this assertion, note that $n\le N$ since *N* is the total number of jumps whereas *n* is the number of jump times. Thus it is reasonable to take $N-n$ to measure the “burstiness” of the walk: for a fixed *N*, the growth of $N-n$ corresponds to the same number of jumps being concentrated at fewer times. As Proposition 4.11’s lower bound decreases with *n*, it also increases with $N-n$.

#### Proof

The upper bound on the distance between endpoints follows from the fact that the walk-with-jumps path of Definition 1.5 is a broken geodesic joining *o* to the walk’s endpoint, of total length $sT + \ell \cdot \sum _i j_i$. This also implies that the distance between any two points on the path is bounded above by their distance *along* the path, ie. the difference between their parameter values if it is parametrized piecewise by arclength as in the Definition. This implies the upper bound needed for quasigeodesicity in Definition 4.7.

To prove the Proposition’s lower bound on the distance between endpoints we sequentially apply Lemma 4.9 and Corollary 4.2, first to the triangle with vertices *o*, $p_i$, and $q_i$ then to the triangle with vertices *o*, $q_i$, and $p_{i+1}$, for *i* increasing from *i* to *n*. (For $i=n$ we interpret “$p_{i+1}$” to refer to the walk’s endpoint.) From these we obtain inductively that for each *i*, the distance from *o* to $q_i$ is at least $st_i + \ell \cdot \sum _{k=1}^i j_k - (2i-1)\delta _{\pi -\theta }$, and from *o* to $p_{i+1}$, at least $st_{i+1}+ \ell \cdot \sum _{k=1}^i j_k - 2i\delta _{\pi -\theta }$ (where for $i = n$ we interpret “$t_{i+1}$” as *T*).

For arbitrary *x* and *y* on the walk-with-jumps path, an entirely analogous argument shows that the distance from *x* to *y* is at least $d_0 - k\delta _{\pi -\theta }$, where $d_0$ is the length of the sub-path bounded by *x* and *y* and *k* is the number of $p_i$ and $q_i$ that lie within this sub-path. The sub-path bounded by *x* and *y* contains least $\frac{k-2}{2}$ jump segments, with the lower bound attained only if *x* and *y* each lie in separate jump segments. Thus using that $d_0 > \frac{k-2}{2}\ell$, we have

$$\begin{aligned} d(x,y) \ge d_0 - \frac{k-2}{2}(2\delta _{\pi -\theta }) - 2\delta _{\pi -\theta } \ge d_0\left( 1 - \frac{2\delta _{\pi -\theta }}{\ell }\right) - 2\delta _{\pi -\theta }. \end{aligned}$$

The right-hand quantity in parentheses above is exactly $\lambda$ from (8), so we have proved the needed lower bound for quasigeodesicity.$\square$

#### Corollary 4.13

Let $(o,\textbf{v},s,\ell ,\theta )$ be the data of a walk with jumps model. Supposing that $\delta _{\pi -\theta } < \ell /2$, for $\delta$ as in Lemma 4.9, define $\lambda$ as in (8). Then for any natural numbers *M* and *N* with $M<\lambda N$, and any fixed $T>0$, no two walks with jumps in the model that share a duration *T* have the same endpoint if the first has *M* jumps and the second, *N*.

The Corollary follows directly by comparing Proposition 4.11’s upper bound for the walk with *M* jumps to the lower bound for the walk with *N* jumps, and applying the fact that $M<\lambda N$ and $n\le N$ to show that the endpoint of the walk with *M* jumps is closer to *o* than the endpoint of the walk with *N* jumps.

As $\ell \rightarrow \infty$ in (8), $\lambda \rightarrow 1$. Therefore, if $\ell$ is chosen to be sufficiently long (relative to $\theta$), then Corollary 4.13 implies that walks with jumps paths with different numbers of jumps have distinct endpoints.

## A criterion for having separate endpoints

In this section we prove Theorem 1.8 from the introduction. This will require the following trigonometric lemma:

### Lemma 5.1

Let $(o,\textbf{v},s,\theta =\pi /2,\ell )$ be the data of a walk with jumps model. For a walk with jumps in the model that has *n* jumps and burst vector $(1,\hdots ,1)$, number the vertices of the associated walks-with-jumps path $p_i$ and $q_i$ as in Definition 1.5. For any $i<n$ such that $t_{i+1}-t_i \ge R$, the angle $\eta _i$ measured at the jump endpoint $q_i$, from the walk axis emanating from $q_i$ to the geodesic arc that joins $q_i$ to the next jump endpoint $q_{i+1}$ satisfies:

$$\begin{aligned} \sin \eta _i \le \frac{\sinh \ell }{\sqrt{\cosh ^2 (sR)\cosh ^2\ell - 1}}. \end{aligned}$$

**Fig. 8:**
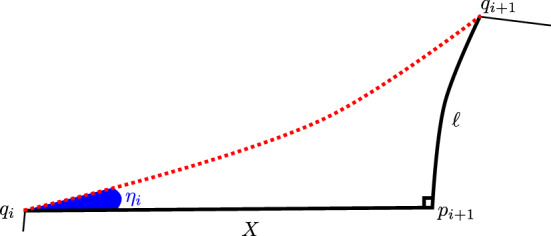
A right triangle

### Proof

The arcs in question emanating from $q_i$ form a right triangle, together with the $i+1$st jump axis, see Figure 8, whose base has length $X = s(t_{i+1}-t_i)$. The length *H* of the triangle’s hypotenuse satisfies $\cosh H = \cosh X\cosh \ell$, by the hyperbolic law of cosines, and by the hyperbolic law of sines the angle $\eta _i$ satisfies $\sin \eta _i = \sinh \ell /\sinh H$. Rewriting the first formula in terms of $\sinh H$ and substituting into the second yields the one given in the Lemma, but with equality and with *X* replacing *sR*. Applying monotonicity of the resulting formula gives the result. $\square$

We now recall the relevant definitions, also from the Introduction.

### Definition 5.2

For $\epsilon > 0$, two walks with jumps in the same walk with jumps model that share a duration $T>0$ are *distinct to resolution* $\epsilon$ if their sequences of jump times $(s_1,\hdots ,s_m)$ and $(t_1,\hdots ,t_n)$ satisfy:

1. for each $i\le \min \{m,n\}$, either $s_i = t_i$ or $|s_i-t_i|>\epsilon$; and
2. there exists some *i* such that $s_i\ne t_i$, or $m\ne n$.

For $R_{min}>0$, a walk with jumps has *minimum refractory length* $R_{min}$ if its burst vector is $(1,\hdots ,1)$—ie. it has no bursts—and for $i\ne j$, $|t_i-t_j|\ge R_{min}$.

### Theorem 1.8

Given $R_{\min }>0$, the parameters $(s,\theta =\pi /2,\ell )$ of a walk with jumps model, and a duration $T>0$, let

$$\begin{aligned} \epsilon = \frac{1}{s}\cosh ^{-1}\left( \frac{\cosh (sR_{min})+1}{\cosh (sR_{min})-\tanh ^2\ell } \right) . \end{aligned}$$

Any two walks with jumps in the given model, having duration *T* and minimum refractory length $R_{min}$, that are distinct to resolution $\epsilon$, have distinct endpoints.

We first prove a special case of Theorem 1.8, from which the general result will follow. It refers to the *walk-with-jumps paths* from Definition 1.5.

### Lemma 5.3

Let $R_{\min }>0$, the data $(o,\textbf{v},s,\theta =\pi /2,\ell )$ of a walk with jumps model, and a duration $T>0$ be given, and define $\epsilon$ as in Theorem 1.8. For a walk with jumps in the model having the given duration, and minimum refractory length $R_{\min }$, which is distinct to resolution $\epsilon$ from a walk having its first jump at time 0, the former walk’s walk-with-jumps path does not intersect the latter walk’s second walk axis (from Definition 2.6). In particular, the two walk-with-jumps paths intersect only at *o*.

**Fig. 9:**
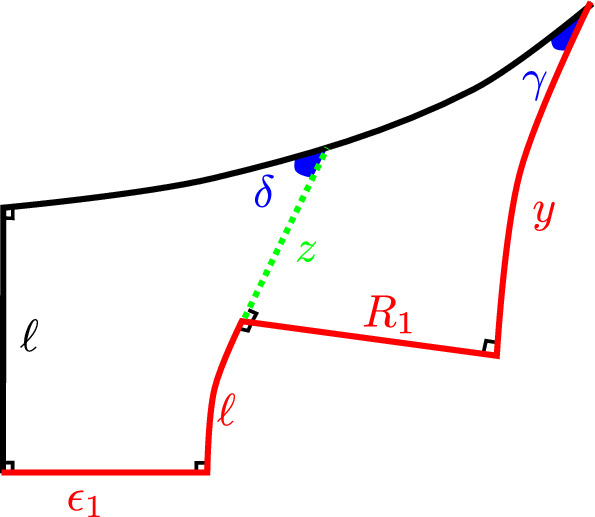
A non-convex hexagon

### Proof

Consider two walks-with-jumps having minimum refractory length $R_{min}$, such that the first has a jump at time 0 and the second has its first jump at time $\epsilon$ and its second at time $\epsilon +R_{min}$, the earliest possible. Then the initial segments of their walk-with-jumps paths belong to sides of the hexagon pictured in Figure 9: those of the first belonging to the black sides, and those of the second, the red. Here $\epsilon _1 = s\epsilon$ and $R_1 = sR_{min}$. As in the Figure, we let *z* be the length of the arc in the second walk’s first jump axis joining the endpoint of its first jump segment to the first walk’s walk axis. Hyperbolic trigonometry gives the following relations between the lengths of the sides of the resulting “Lambert quadrilateral” (one with three right angles) and the angle $\delta$:

$$\begin{aligned} \tanh (\ell +z) = \cosh \epsilon _1 \tanh \ell ,\quad \hbox {and}\quad \cos \delta = \sinh \ell \sinh \epsilon _1. \end{aligned}$$

As in the proof of Proposition 3.2 we note that if the product $\sinh \ell \sinh \epsilon _1$ were to exceed 1, it would simply mean that the first walk’s first walk axis and the second walk’s first jump axis did not intersect. This would simultaneously imply that $\cosh \epsilon _1\tanh \ell > 1$, and hence that the left-hand equation above was also not satisfied.

However we aim to choose $\epsilon$ small enough that this does not occur; but large enough that the quantity *y* of Figure 9 is at least $\ell$. To do this we apply angle-sum identities and simplify to solve the left-hand equation above for *z*:

$$\begin{aligned} \tanh z = \frac{\cosh \epsilon _1-1}{\coth \ell - \tanh \ell \cosh \epsilon _1}. \end{aligned}$$

We now observe that since the angle $\delta$ is less than $\pi /2$, there is a Lambert quadrilateral contained in the hexagon of Figure 9 that has its side of length $R_1$ as one side, a side intersecting that with length *z*, and another side properly contained in the one with length *y*. Applying the same trigonometric law to this quadrilateral, we obtain:

$$\begin{aligned} \tanh y \ge \cosh R_1\tanh z = \frac{\cosh R_1(\cosh \epsilon _1-1)}{\coth \ell - \tanh \ell \cosh \epsilon _1}. \end{aligned}$$

Thus to ensure that *y* is at least $\ell$, it is enough to choose $\epsilon$ so that the right-hand side above is at least $\tanh \ell$. Setting it equal and solving for $\epsilon$ yields the value stated in the theorem.

We note that the right-hand lower bound for *y* above is increasing in each of $\epsilon _1$ and $R_1$. This implies that if the second walk’s first jump occurs at a time *at least* the value of $\epsilon$ stated in the theorem, then its second jump will not cross the walk axis of the first walk with jumps, since this second jump occurs at a time at least $R_{min}$ after that of the first. We also note that the angle labeled $\gamma$ in Figure 9, between the first walk’s walk axis and the second walk’s second jump axis, is less than the angle labeled $\delta$ in the figure. This is because the quadrilateral in the figure, with angles $\gamma$, $\pi -\delta$, and two right angles, has angle sum less than $2\pi$.

Let $q_i$ be the endpoint of the *i*th jump of the second walk, and let $\tau _i$ be the geodesic ray starting at $q_i$ and passing through $q_{i+1}$. Since the difference in jump times $t_{i+1}-t_i$ is at least the minimum refractory length $R_{min}$, and $R_1 = sR_{min}$, Lemma 5.1 asserts that the angle $\eta _i$ at $q_i$ made by $\tau _i$ and the walk axis emanating from $q_i$ satisfies:

$$\begin{aligned} \sin \eta _i \le \frac{\sinh \ell }{\sqrt{\cosh ^2 R_1\cosh ^2\ell - 1}}. \end{aligned}$$

We claim first that this bound implies that $\eta _i\le \pi /2-\delta$; or equivalently, comparing the bound with the formula for $\cos \delta$, that $\sinh \epsilon _1\sqrt{\cosh ^2 R_1\cosh ^2\ell - 1}\ge 1$. Substituting gives the left-hand side:

9 $$\begin{aligned} \sqrt{\left[ \left( \frac{\cosh R_1+1}{\cosh R_1 - \tanh ^2\ell }\right) ^2 - 1\right] \left( \cosh ^2 R_1\cosh ^2\ell - 1\right) } \end{aligned}$$

It is a straightforward computation to show that (9) exceeds 1.

We now claim that as a consequence of this, the first and second walks with jumps do not cross.

Let $\rho$ be the walk axis of the first jump in Figure 9 and let $\lambda _i$ be the *i*th jump axis of the second walk. We claim that if $\lambda _i$ intersects $\rho$, the intersection between $\lambda$ and $\rho$ in the quadrant bounded by $\lambda$ and $\rho$ containing *o* is at an angle $\xi _i\le \delta$. Indeed, if $\lambda _i$ does not intersect $\rho$, then for all $j\ge i$, $\lambda _j$ does not intersect $\rho$ because $\lambda _i$ separates $\lambda _j$ from $\rho$, by Corollary 4.5. If $\lambda _{i-1}$ and $\lambda _i$ intersect $\rho$, then using the walk axis between $\lambda _{i-1},\lambda _i$, there is a quadrilateral whose angles are $\pi -\xi _{i-1},\,\frac{\pi }{2},\,\frac{\pi }{2}$ and $\xi _i$ since the angle sum must be less than $2\pi$, we have $\xi _i\le \xi _{i-1}$. Since $\xi _1=\delta$, $\xi _i\le \delta$ by induction.

**Fig. 10:**
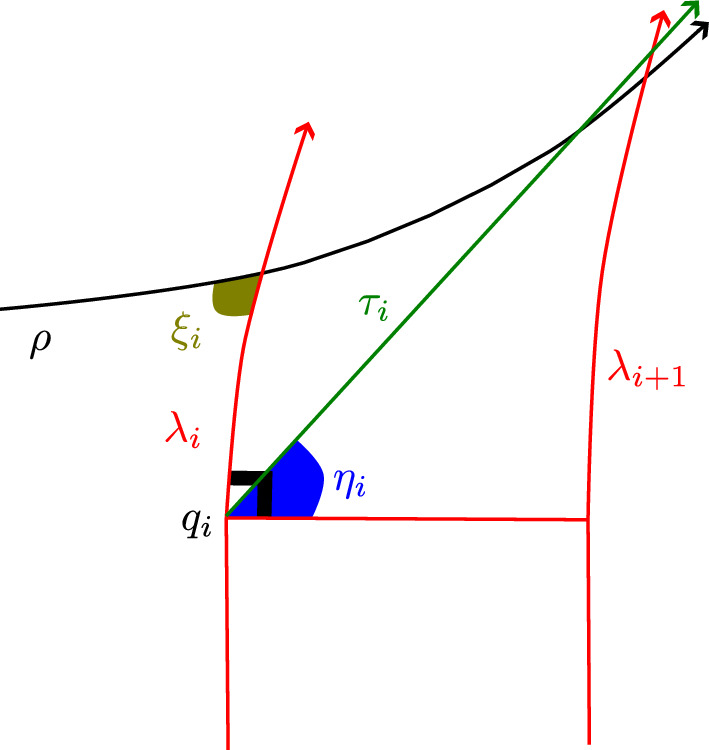
The setup for showing that the first and second walk do not intersect

Aside from its first jump, the first walk travels entirely above $\rho$, see Corollary 4.5 for details. Therefore, if the first and second walks with jumps cross, the second walk must cross $\rho$. Since $\eta _i$ is acute, if $q_i$ sits below $\rho$, the ray following the walk axis at $q_i$ heading in the direction of travel cannot cross $\rho$. Thus if the first and second walks with jumps cross, then there exist a pair $q_i,q_{i+1}$ that lie on opposite sides of $\rho$ since the second walk must intersect $\rho$ in a jump. Then $\tau _i$ crosses $\rho$. However we observe that if $\tau _i$ and $\rho$ cross, then there would be a triangle formed by $\rho$, $\tau _i$ and $\lambda _i$ where two of the interior angles are $\frac{\pi }{2}-\eta _i$, $\pi -\xi _i$ (Fig. 10). We have $\frac{\pi }{2}-\eta _i\ge \frac{\pi }{2} - (\frac{\pi }{2}-\delta ) = \delta$, and $\pi -\xi _i\ge \pi -\delta$, so the interior angle sum of this triangle is at least $\pi -\delta +\delta = \pi$ so the third vertex of this triangle at the intersection of $\tau _i$ and $\rho$ must be ideal. Therefore $\rho ,\tau _i$ do not cross, so the first and second walks with jumps cannot intersect except at *o*. $\square$

Theorem 1.8 will now follow directly from the more precise result below.

### Theorem 5.4

Suppose $(s_1,\hdots ,s_m)$ and $(t_1,\hdots ,t_n)$ are the jump time sequences of two walks with jumps satisfying the hypotheses of Theorem 1.8. Define

$$\begin{aligned} k_0 = \max \left( \{1\}\cup \{k\,|\,s_i = t_i\ \hbox {for all}\ i<k\}\right) , \end{aligned}$$

The intersection of the corresponding walk-with-jumps paths is the union of their $i^\textrm{th}$ walk and jump segments, for all $i< k_0$, together with the shorter of the two $k_0^\textrm{th}$ walk segments.

### Proof

If (say) $s_1 = 0$ and $t_1>0$ then $k_0$ defined as above equals 1, the first walk-with-jumps path has a degenerate first walk segment consisting only of the origin *o*, and the Theorem’s conclusion specializes to that of Lemma 5.3. Our goal is to reduce to this case.

Let us assume now that either $m>n$ and $s_i = t_i$ for all $i\le n$, whence $k_0 = n+1$, or that $0<s_{k_0}<t_{k_0}$. Then for the two walk-with-jumps paths, constructed as in Definition 1.5, the points $p_i$ and $q_i$ coincide for all $i<k_0$. Moreover, the $k_0^\textrm{th}$ walk segment of the walk with jump times $s_i$ is a subsegment of the other walk’s $k_0^\textrm{th}$ walk segment. Let $\gamma _{k_0}$ and $\lambda _{k_0}$ be the $k_0^\textrm{th}$ walk and jump axes, respectively, of the walk with jump times $s_i$, defined as in 2.6. By Lemma 2.7, these are characterized by containing this walk’s respective walk and jump *segments*. We claim that for the half-planes $H_{k_0-1}^-$ and $K_{k_0}^{-}$ respectively bounded by these axes, identified in Corollary 4.5, $H_{k_0-1}^-\cap K_{k_0}^{-}$ contains the union of the segments that were identified above as common to the two walk-with-jumps paths, and the intersection of complementary half-spaces $H_{k_0-1}^+\cap K_{k_0}^+$ contains the remainder of each of the two walk-with-jumps paths.

Corollary 4.5 asserts in particular that $H_{k_0-1}^-$ contains the duration-$s_{k_0-1}$ initial segment, and $K_{k_0-1}^-$ the duration-$s_{k_0}$ initial segment, of the walk-with-jumps path corresponding to the walk with jump times $s_i$. We note that $H_{k_0-1}^-$ also contains this walk’s $k_0^\textrm{th}$ walk segment, since this segment lies in the bounding geodesic $\gamma _{k_0}$. Therefore it contains all of the common segments identified above, and the duration-$k_0$ initial segment does as well.

The remainder of the walk-with-jumps path corresponding to the walk with jump times $s_i$, outside the union of its segments in common with the other walk-with-jumps path, consists of the union of its $k_0^\textrm{th}$ jump segment with the complement (in the path) of the duration-$s_{k_0}$ initial segment. It is contained in the complement (in the path) of the duration-$s_{k_0-1}$ initial segment, so by Corollary 4.5, it is contained in $H_{k_0-1}^+$. The Corollary moreover asserts that $K_{k_0}^+$ contains the remainder of the path beyond the duration-$s_{k_0}$ initial segment. Since the $k_0^\textrm{th}$ jump segment is contained in the bounding geodesic $\lambda _{k_0}$ of $K_{k_0}^+$, it too contains the entire remainder of the path outside the union of its common segments with the other path.

Turning attention to the walk-with-jumps path corresponding to the walk with jump times $t_i$, we note that the half-planes $H_{k_0-1}^{\pm }$ defined for the first path play the same role for this path as for that one: $H_{k_0-1}^-$ contains the union of its duration-$t_{k_0-1}$ initial segment with its $k_0^\textrm{th}$ walk segment, and $H_{k_0-1}^+$ contains the complement (in the path) of the duration-$t_{k_0-1}$ initial segment. It follows that $H_{k_0-1}^-$ contains the union of segments that the first path has in common with this one, and that $H_{k_0-1}^+$ contains the complement in this path of that union of segments.

The half-planes $K_{k_0}^{\pm }$ defined for the first path do not play the same role for this path as for that one; however, their bounding geodesic $\lambda _{k_0}$ intersects this walk’s $k_0^\textrm{th}$ walk segment at an angle of $\theta$ in its interior, between its points of intersection with this walk’s $k_0-1^\textrm{st}$ and $k_0^\textrm{th}$ jump axes—its endpoints. Arguing as in Lemma 4.1 we therefore find that $\gamma _{k_0}$ is disjoint from each of these jump axes, so by Corollary 4.5$K_{k_0}^-$ contains the duration-$t_{k_0-1}$ initial segment and $K_{k_0}^+$ contains the complement (in the path) of the duration-$t_{k_0}$ initial segment. Moreover, $\gamma _{k_0}$ divides the first walk’s $k_0^\textrm{th}$ walk segment from its complement in the second walk’s $k_0^\textrm{th}$ walk segment. Therefore $K_{k_0}^+$ contains the complement in the second walk’s walk-with-jumps path of the union of common segments. This establishes the claim’s second half.

The claim implies that if the first walk’s walk-with-jumps path has a point of intersection with that of the second outside the union of common segments, then this point of intersection must belong to the complement of the union of common segments in the second walk-with-jumps path as well. This is because these complementary sub-paths are walled off from the union of common segments within the quadrant $H_{k_0-1}^+\cap K_{k_0}^+$. We now observe that these complementary sub-paths are themselves walk-with-jumps paths of two related walks with jumps, to which we will apply Lemma 5.3 to finish the proof.

Let $p_{k_0}$ be as in Definition 1.5 for the first walk’s walk-with-jumps path, ie. the terminal point of its $k_0^\textrm{th}$ walk segment, and let $\textbf{w}$ be the outward-pointing tangent vector to the $k_0^\textrm{th}$ walk path at $p_{k_0}$. Then $p_{k_0}$ may be taken as the common origin, and $\textbf{w}$ the common initial vector, for two walks with jumps that otherwise share the given walks’ initial data, each have duration $T-s_{k_0}$, and whose jump times are $(s'_{k_0}=0,s'_{k_0+1},\hdots ,s'_m)$ and $(t'_{k_0},\hdots ,t'_{n})$, respectively, where $s'_j = s_j - s_{k_0}$ for $k_0\le j\le m$ and similarly for $t'_j$. The walk-with-jumps paths associated to these two walks are exactly the portions of the original walks’ walk-with-jumps paths complementary to the union of their duration-$T_0$ initial segments, for all $T_0<s_{k_0}$. The two new walks satisfy all hypotheses of Lemma 5.3, so that result’s conclusion implies that their walk-with-jumps paths intersect only at the origin $p_{k_0}$. The current result follows. $\square$

We finally return to the setting of Example 1.11, using Theorem 5.4 to embed a binary tree in $\mathbb {H}^2$ so that paths in the tree are taken to walk-with-jumps paths.

### Corollary 5.5

For a given $R_{\min }>0$ and the data $(o,\textbf{v},s,\theta =\pi /2,\ell )$ of a walk with jumps model, define $\epsilon = \epsilon (R_{\min },s,\ell )$ as in Theorem 1.8, and let $m = \max \{R_{\min },\epsilon \}$. For a given $n\in \mathbb {N}$ and a binary tree $\mathcal {T}$ with root vertex $p_0$, there is an embedding of a finite subtree of $\mathcal {T}$ to $\mathbb {H}^2$, taking $p_0$ to *o*, with the property that the path in $\mathcal {T}$ encoded by any binary sequence $w = w_1\hdots w_n$ as in Example 1.11 (with each $w_i\in \{0,1\}$) maps to the walk-with-jumps path of a walk with jumps having the given initial data and duration $T\doteq n\cdot m$, with a jump at time *t* if and only if $t=(i-1)m$ for some *i* such that $w_i=1$.

### Proof

We recall from Example 1.11 that $w=w_1\hdots w_n$ encodes a path in $\mathcal {T}$ as follows: at the root proceed left if $w_1=1$ and right if $w_1=0$. At the $(i-1)$th vertex of the path, proceed left if $w_i=1$ or right if $w_i=0$. We will map this path to the walk-with-jumps path of the walk with the given initial data and jump time sequence $((i_1-1)m,\hdots ,(i_k-1)m)$, where $i_1< \hdots < i_k$ is the set of $i\in \{1,\hdots ,n\}$ such that $w_i = 1$.

Note that such a walk has $k+1$ walk segments, where *k* is the number of indices *i* such that $w_i = 1$; and for $1< j\le k$, the $j^\textrm{th}$ walk segment has length $s(i_j-i_{j-1})m$. (And the first has length $s(i_1-1)m$, and the last, $s(n-i_k+1)m$.) The idea is to divide each walk segment into equal-length subsegments of length *sm*, then map the path encoded by *w* via a continuous map taking $p_0$ to *o*, such that the $i^\textrm{th}$ edge of the path in $\mathcal {T}$ maps to one of these subsegments if $w_i=0$ (ie. $i\ne i_j$ for any $j\le k$) and to the union of a single jump segment with the subsequent walk sub-segment if $w_i=1$. We prescribe that each edge of the path in *T* have length 1 and require the restriction to each edge to have constant speed.

Let $\mathcal {T}_0$ be the union of the paths in $\mathcal {T}$ encoded by all binary sequences of length *n*, a finite subtree. There is a well-defined map on $\mathcal {T}_0$ that restricts on each such path to the one defined in the paragraph above: for any point $p\in \mathcal {T}_0$ there is a unique embedded edge path in $\mathcal {T}_0$ joining $p_0$ to *p* (since $\mathcal {T}_0$ is a tree), and for any two paths encoded by binary sequences containing this as a sub-path, the maps to the corresponding walk-with-jumps paths agree on the sub-path, by Theorem 5.4 and the canonical nature of the map’s construction above.

Note that the walk with jumps corresponding to any path encoded by a binary sequence has minimum refractory length $R_{\min }$, since $m\ge R_{\min }$. And any two distinct such walks are distinct to resolution $\epsilon$, since $m\ge \epsilon$. It therefore follows again from Theorem 5.4 that the map on $\mathcal {T}_0$ is an embedding. $\square$

## Further Directions

The above results establish some of the basic features of walks with jumps, but leave open a number of directions for further biological and mathematical exploration.

### Biological explorations: empirical tests

The framework developed here enables empirical testing of the applicability of the walks-with-jumps model to neural coding, both for decision-making and the representation of perceptual spaces.

A test of the applicability to decision-making or classification could be carried out on a set of spike trains recorded during a paradigm in which the experimental animal makes a binary decision, such as the classic dataset of responses to random-dot motion in area MT of the macaque (Britten et al. (1993)). For a given set of parameters of a walk-with-jump model, each spike train has a definite trajectory. These trajectories fall into two classes, as specified by the animal’s binary decision. One can then ask the extent to which their endpoints form separate clusters, in the geometry of $\mathbb {H}^2$. A convenient yardstick for this purpose is leave-one-out decoding accuracy: each spike train can be regarded as unknown, and then decoded as belonging to the cluster for which it has the smallest mean distance to the cluster members. This decoding accuracy can then be examined as a function of the parameters of the walk-with-jump model. Note that, in the limit of a jump angle of zero, decoding based on the walk with jump model is equivalent to decoding based on spike count: all walks remain in the direction of the unit tangent vector $\textbf{v}$, at a distance equal to $sT+N$ from the origin.

Testing applicability to representation of perceptual spaces is also possible, and is motivated by evidence that in some domains, perceptual similarity indicates an underlying hyperbolic geometry, as shown by Zhou et al. (2018). If this perceptual geometry is generated by the dynamics of neural activity as modeled here, then the endpoints of walks-with-jumps should recapitulate the similarity structure of the space. We note that hyperbolic characteristics of the hippocampal map of space have also been identified (Huanqiu Zhang et al. 2023). In this case, the hyperbolic characteristics can be explained by the spatial characteristics of neural selectivity, but the possibility of a contribution of spike train dynamics has not been investigated.

### Mathematical explorations: comparing point-process distances

A walk with jumps model yields a number of natural distances that can be defined between samples of point processes other than $\mathbb {H}^2$-distance between their endpoints, such as the Hausdorff distance between the paths. It is of interest to understand these distances’ relationships to each other and to others in the literature, e.g. the edit-length distance between spike trains (Victor and Purpura 1997).

While the precise relationships between these distances are at present unclear, some distinctions are evident. Specifically, Proposition 3.2 here exhibits, for any walk-with-jumps path with a single jump and sufficiently large duration *T*, another path in the same walk with jumps model that has the same endpoint but a number of jumps that increases linearly with *T*. Thus, although the $\mathbb {H}^2$ distance between the endpoints of these two spike trains is zero, the edit-length distance between them is bounded below by an amount that increases linearly in *T*. This is important, as it demonstrates that the walk-with-jumps model is, in fact, not merely a recasting of the edit-length distance.

Regarding the relationship between the endpoint and Hausdorff distances in the walk with jumps model, we first observe that by the results of Section 3 these distances are not equivalent, since there are walks with jumps having identical endpoints but substantially different jump times—hence a non-zero Hausdorff distance between the corresponding walk-with-jumps paths. Hausdorff distance is difficult to compute precisely, since it depends in a sense on knowing (or at least bounding) the distance between any pair of points belonging to the different paths. However, our results plus standard facts of hyperbolic geometry imply—perhaps surprisingly—that the Hausdorff distance between walk with jumps paths is bounded above, additively, in terms of the distance between their endpoints.

#### Proposition 6.1

Let $(o,\,\textbf{v},\,s,\,\theta ,\,\ell )$ be the data of a walk with jumps model such that $\delta _{\pi -\theta } < \ell /2$, for $\delta _{\theta } = \ln \left( \frac{2}{1-\cos \theta }\right)$ as in Lemma 4.9. Suppose $\sigma _1,\sigma _2$ are the walk with jumps paths of two walks with jumps in the model, with the property that the distance between the endpoints of $\sigma _1$ and $\sigma _2$ is at most $d_e\ge 0$. Then there exists $D = D(\ell ,\delta _{\pi -\theta })$— depending only on the model’s parameters—so that the Hausdorff distance between $\sigma _1,\sigma _2$ is bounded above by $D+d_e$.

#### Proof

By Proposition 4.11, there exist $\lambda \ge 1$ and $\epsilon \ge 0$ so that $\sigma _1,\sigma _2$ are $(\lambda ,\epsilon )$–quasigeodesics where $\lambda ,\epsilon$ depend on $\ell ,\theta$. Let $\gamma _1,\gamma _2$ be geodesics between *o* and the endpoints of $\sigma _1,\sigma _2$ respectively. By “quasigeodesic stability” (see eg. (Bridson and Haefliger (1999), Theorem III.H.1.7)), there exists $D_0$ depending on $\lambda ,\epsilon$ so that the Hausdorff distance between $\sigma _i$ and $\gamma _i$ is bounded above by $D_0$.

The Hausdorff distance between $\gamma _1$ and $\gamma _2$ is equal to the hyperbolic distance $d_e$ between their non-shared endpoints. This follows for instance from the hyperbolic law of cosines, which asserts that $\cosh c = \cosh a\cosh b - \sinh a\sinh b\cos \theta$, where *a*, *b*, and *c* are the sidelengths of a triangle and $\theta \in (0,\pi )$ is the angle opposite *c*. If we take *b* to be the distance from *o* to a fixed point on $\gamma _1$, say, and let $a(t) = t$ be the distance from *o* to the point *x*(*t*) on $\gamma _2$ at distance *t* away from it, the law of cosines above shows that $c=c(t)$ is a convex function of *t* and hence attains its maximum at the far endpoint. Thus the far endpoint of $\gamma _2$ is furthest from any given point of $\gamma _1$; applying the same argument with roles reversed shows that the far endpoint of $\gamma _2$ is furthest from it.

Let $D = 2D_0$. By the triangle inequality for the Hausdorff distance,

$$\begin{aligned} d_{\text {Hausdorff}}(\sigma _1,\sigma _2)&\le d_{\text {Hausdorff}}(\sigma _1,\gamma _1) + d_{\text {Hausdorff}}(\gamma _1,\gamma _2) + d_{\text {Hausdorff}}(\gamma _2,\sigma _2) \\ &\le D_0 + d_e+D_0 = D +d_e. \end{aligned}$$

$$\square $$

Here are two extensions that we have investigated but do not have clear answers to:

1. For what initial data and at what scale is the bound $D = D(\ell ,\delta _{\pi -\theta })$ on Hausdorff distance between walks-with-jumps with the same endpoints effective relative to the overall length of the associated walks-with-jumps paths? Compounding this problem is the fact that the value *D* can be hard to estimate. For example, the proof of (Bridson and Haefliger (1999), Theorem III.H.1.7) shows that *D* exists and provides an indirect method to compute *D* but there is no explicit formula for a good estimate.
2. Can differences in walk data predict the Hausdorff distance between two walks with jumps? Specifically, what differences cause the Hausdorff distance between two walks with jumps to exceed the bound from Proposition 6.1? An answer would imply another “point of no return” result.

### Other Directions

Another direction for further exploration is the structure of the endpoint set for walks with jumps of a fixed duration and number of jumps. It is connected, but its shape remains to be characterized.

Biological considerations suggest other directions for study and extensions. Neural spike trains are often considered to be samples from a point process; it is therefore of interest to determine how the point process model (e.g., Poisson, Poisson with refractory period, Markov, etc.), impacts the distribution of endpoints and the extent to which walks with jumps may cross. Finally, just as the activity of a single neuron can be formalized as a point process, multineuronal activity can be formalized as a labeled point process. In analogy with the walk-with-jump model of a single neuron’s activity, multineuronal activity could then be modeled as a walk with jumps in a higher-dimensional space, possibly hyperbolic or with hyperbolic subspaces, in which each neuron’s activity corresponds to a different kind of jump.

## Data Availability

There are no data associated with this paper.
